# Applying human-centered design to adapt a multifaceted implementation strategy for integrating HIV and NCD services in Lusaka, Zambia: Healthcare worker perspectives

**DOI:** 10.1371/journal.pgph.0005879

**Published:** 2026-02-02

**Authors:** Tulani Francis L. Matenga, Michael E. Herce, J. Hope Corbin, Joseph Mumba Zulu, Chilambwe Mwila, Chomba Mandyata, Christiana Frimpong, Mmamulatelo Siame, Perfect Shankalala, Namwinga Nachalwe, Peter Mbewe, Peter Chisenga, Pendasambo Sichone, Maurice Musheke, Wilbroad Mutale, Oliver Mweemba

**Affiliations:** 1 Department of Health Promotion and Education, School of Public Health, University of Zambia, Lusaka, Zambia; 2 Centre for Infectious Disease Research in Zambia (CIDRZ), Lusaka, Zambia; 3 Institute for Global Health and Infectious Diseases, University of North Carolina, Chapel Hill, North Carolina, United States of America; 4 Department of Health and Community Studies, Western Washington University, Bellingham, Washington, United States of America; 5 Department of Health Policy and Management, School of Public Health, University of Zambia, Lusaka, Zambia; 6 Department of Paediatrics and Child Health, School of Medicine, University of Zambia, Lusaka, Zambia; University of California San Francisco, UNITED STATES OF AMERICA

## Abstract

There has been limited research reporting approaches used in the adaptation of implementation strategies for integrating HIV and NCD services that involve healthcare providers in low- and middle-income country (LMIC) settings. This paper describes how human-centered design (HCD) was used to 1) adapt a multifaceted implementation strategy for integrating HIV and NCD services through end-user engagement and 2) share insights from the specific suggestions made through the HCD process with healthcare providers. As part of the TASKPEN an implementation science-driven intervention aimed at task shifting and integrating the evidence-based WHO Package of Essential NCDs Interventions (WHO-PEN) approach into routine healthcare settings for PLHIV, we co-designed implementation strategy components (i.e., “sub-components”) to strengthen HIV/NCD integration in four HIV clinics in Lusaka, Zambia. The HCD process involved qualitative approaches with healthcare providers, namely lay and non-physician healthcare workers (NPHWs) such as nurses and community healthcare workers (CHWs). A four-phased approach of exploration through formative qualitative work, ideation with intervention deliverers, analysis, and refinement was used to develop strategy sub-components. Rapid thematic data analysis was used to synthesise the data. Applying HCD to a locally-informed intervention for screening and managing cardio-metabolic co-morbidities for Zambian PLHIV informed the addition of sub-components to the multi-faceted implementation strategy to improve adoption, appropriateness, and feasibility. Through co-design workshops, healthcare providers suggested specific sub-components be added to enhance the intervention. Specifically, four sub-component strategy ideas were produced: introducing NCD/HIV champions, circulating a weekly facility NCD medication bulletin, dashboard reporting of HIV/NCD cases, and community sensitization. The use of HCD in implementation science is a promising approach to refining implementation strategies that are likely to result in the success of HIV/NCD integration in Zambia and similar LMIC settings.

## Introduction

HIV has become a chronic and manageable disease for persons receiving life-saving antiretroviral therapy (ART). As more people living with HIV (PLHIV) are living longer on treatment, they face an increased risk of HIV-associated non-communicable diseases (NCDs), including cardiovascular disease, cancer, and metabolic disorders such as diabetes [1]. As of 2022, 39 million people globally were living with HIV, and out of these, 29 million were accessing ART [2]. Sub-Saharan Africa (SSA) alone endures the highest burden of HIV, with 70% of the global HIV burden [2]. The increasing incidence of NCDs among PLHIV is driven by several factors which include an aging PLHIV population, NCD risk factors compared to those without HIV, the direct inflammatory effects of HIV infection, lifestyle factors such as tobacco and alcohol use, and ART exposure [3]. The co-occurrence of HIV and NCDs has thus become a growing challenge in many low- and middle-income countries (LMICs) experiencing this epidemiological transition [4].

Integrating HIV and NCD services can lead to improved health system efficiencies and outcomes for PLHIV [5] by reducing duplication and fragmentation of services [6], identifying undiagnosed NCDs [7], supporting drug and diagnostic equipment procurement, and enhancing treatment support for the management of both conditions [8]. However, the design and implementation of integrated HIV/NCD services in LMICs can be challenging due to resource constraints, inadequate infrastructure, and limited human resources for health capacity [9]. Engaging intervention deliverers such as healthcare workers in creating solutions to NCD care challenges is an approach that may increase ownership and acceptance [10]. Human-centered design (HCD) is an approach that aims to understand the context, generate potential solutions, prototype and test, and iterate based on user feedback [11]. This approach includes, empathise, define, ideate, prototype, and test phases [12] were stakeholders are engaged to gather insights and ensure interventions fit the local context [13, 14]. HCD is essential for sustainable innovation, emphasising empathy and interdisciplinary collaboration [15]. Although HCD is gaining popularity, its use in global health research overall is limited [16].

We co-designed implementation strategy components (i.e., “sub-components”) of the TASKPEN package designed to manage cardiovascular disease risk factors and the cardiometabolic complications of HIV among PLHIV (Table 1). The TASKPEN package included: 1) a one-stop shop for HIV/NCD screening, diagnosis, treatment, and care implemented through reconfiguration of patient and work flows in ART and differentiated service delivery (DSD) clinics; 2) use of WHO-PEN guidelines and training materials adapted to the Zambian setting through the provision of adapted training and educational materials, support to healthcare workers, and task-shifting integrated NCD management to NPHWs such as nurses and CHWs, building on existing task-shifting practices used in routine HIV care; 3) use of an NCD-focused module added to the national HIV Electronic health recode (EHR) with integrated decision support and regular collaborative data review meetings among frontline health workers focused on EHR-derived dashboard indicators to identify and act on areas for quality improvement; 4) cardio-metabolic NCD diagnostic testing and monitoring through provision of basic NCD laboratory and vital sign equipment; and 5) Strengthened NCD medication supply chain, including multi-month dispensing (MMD), through engagement with Ministry of Health (MOH) medication supply chain structures to build support for NCD MMD in ART clinics and healthcare worker skills acquisition on pharmacy management and MMD [17].

**Table 1:** Overview of evidence-based interventions and multi-faceted implementation strategy sub-components that formed the TASKPEN package as well as relevant operational details.

TASKPEN Evidence-based Interventions	TASKPEN Multi-faceted Implementation Strategy (*Main component and sub-components*)	Relevant Operational Details
**1. One-stop shop for integrated HIV and NCD services**	a. Change/Co-locate service delivery sites (main strategy)	Creating a “one stop shop” enables co-management of HIV and NCDs at the same time and place where PLHIV receive their HIV care. TASKPEN study team leadership support MOH clinical leaders (usually the ART Nurse In-Charge and Medical Superintendent) to reorganise work flows in the ART/DSD clinic to reduce interdepartmental referrals within the facility and streamline NCD services such that they can be provided at each step of the patient journey within the ART clinic to create an Integrated ART/NCD Clinic.
**2. Health worker practice guidelines and training materials based on WHO-PEN**	a. Practice facilitation (sub-component) b. Revise professional roles/ task-shifting and sharing (sub-component)	TASKPEN clinical mentors or “practice facilitators” provide training on clinical algorithms adapted from WHO-PEN and peer-to-peer with health workers of the same cadre to support skill acquisition in the screening, diagnosis, and treatment of hypertension, diabetes, hyperlipidemia, and tobacco use. Fromal skills training is done prior to introduction of the TASKPEN intervention using a combination of didactic lecture and hands-on practice with supportive supervision. Nurses and peer treatment supporters from the ART clinic become NCD providers under the guidance of a NCD “champion” selected by facility management to lead NCD integration, and take on added responsibilities within their practice setting for providing integrated cardiometabolic NCD services. All health workers at the ART/ DSD clinic are supported to deliver the intervention, which typically includes one doctor or clinician, one pharmacist or pharmacy technologist, one laboratory technician, and a handful of nurses and peer treatment supporters.
**3. Electronic Health Record (EHR)**	a. Change record system (sub-component)	TASKPEN Clinical Mentors and the TASKPEN EHR Programmer work with members of the CIDRZ Medical Informatics team, MOH, and implementing partners responsible for the EHR to develop, pilot test, and refine a dedicated module for the HIV EHR (SmartCare) to record cardiometabolic NCD data for PLHIV. The EHR module includes new data fields for NCD diagnoses, NCD-related lab values, NCD medications prescribed, and smoking cessation. The module also supports data aggregation for monitoring of basic performance indicators (e.g., # and % of PLHIV with ≥1 documented blood pressure measurement during reporting period, # and % of PLHIV with documented hypertension who collected ≥1 anti-hypertensive medication dispensation during reporting period, etc.)
**4. Diagnostic testing and monitoring for cardiometabolic NCDs**	a. Change physical structure and equipment (sub-component)	The TASKPEN Laboratory Technologist supports procurement and placement of the ichroma II point-of-care (POC) diagnostic system in the ART/ DSD clinic or adjoining lab to enable one- screening and diagnostic testing for hemoglobin A1c and low-density lipoprotein (LDL) cholesterol. TASKPEN Clinical Mentors coach appropriate ordering, interpretation, and action on test results.
**5. Supply chain strengthening for NCD medications, including multi-month dispensing (MMD)**	a. Creating working groups (sub-component) b. Practice facilitation (sub-component)	The TASKPEN Pharmacist works with MOH counterparts at central and provincial levels to create supply chain working groups and strengthens the existing logistics and supply chain management system to meet needs for antihypertensives, oral hypoglycemic agens and insulin, and statins. TASKPEN Clinical Mentors and MOH NCD Champions based at the clinics support adoption of MMD for NCD medications within the existing PEPFAR program, and signal need for emergency provision of NCD medications by the TASKPEN study in case of medication stock outs.

In this article, we describe the process of using HCD thinking as an approach to refine the TASKPEN intervention package and multi-faceted implementation strategy for integrated HIV/ NCD service delivery, based on the WHO-recommended package of healthcare worker-led treatment algorithms for NCDs [18] and how we prepared health facilities to pilot test TASKPEN. We also share process outcomes and insights of the HCD process to inform future work on the health system and clinical intervention integration in similar settings. The article traces the evolution from the original TASKPEN strategy through formative work to better understand intervention deliverers challenges experienced with provision of NCD care to PLHIV, HCD-driven co-design workshops to find solutions to challenges and iterative refinement, culminating in a final improved implementation package.

## Methods

### Study design

We used a HCD approach to co-design implementation strategy components (sub-components) to strengthen HIV/NCD integration in four HIV clinics in Lusaka, Zambia to achieve the aims of the TASKPEN study. Given that the health sector often faces complex issues and operates under severe constraints, including limited funding and staffing, these environments demand approaches that are flexible, adaptive, and responsive to population needs. We therefore applied a four-stage abridged adaptation of HCD: **Exploration**, **Ideation**, **Analysis** and **Synthesis**, and **Refinement** to obtain user feedback and improve key components of the intervention’s delivery.

In our prior formative work, we conducted qualitative data collection as part of the **Exploration** phase of the HCD process to understand the challenges, needs, and experiences of PLHIV with NCDs. Study activities included key informant interviews (KIIs), in-depth interviews (IDIs) with PLHIV, focus group discussions (FGDs) with healthcare providers namely, community healthcare workers, and non-physician healthcare workers (NPHWs), and onsite observations. Data were collected using semi-structured discussion guides that explored domains adapted from the Consolidated Framework for Implementation Research (CFIR) [19, 20], including complexity, perceived patient needs and resources, perceived healthcare worker challenges, structural characteristics, knowledge and beliefs, planning, engaging, and executing, among others. Questions were open-ended to allow probing for emerging major and minor themes. FGDs lasted approximately 1–2 hours and interviews 20–60 minutes. IDIs, FGDs, and KIIs were audio recorded with participant permission, conducted within the facilities with measures to ensure privacy, and all participants provided written informed consent.

### Study setting

Data collection for the formative qualitative work and the HCD workshops was conducted at four high-volume HIV facilities two level-one (district) hospitals and two primary health centers in Lusaka, Zambia. The selected facilities serve a wide catchment area and a large population of PLHIV, with more than 800 clients seen per month. Level-one hospitals provide medical, surgical, and obstetric care in support of health center referrals, while primary health centers offer essential preventive and health maintenance services such as vaccination, family planning, and antenatal care. These facilities serve both low- and middle-income populations, allowing inclusion of a diverse sample of study participants from varied backgrounds. Study activities were conducted between February 2021 and August 2021.

### Study population and sampling

In the formative qualitative work, we conducted four FGDs with healthcare providers (eight participants in each FGD) and four FGDs with community healthcare workers (eight participants in each FGD). We also conducted 20 IDIs with recipients of care with co-morbidities and six KIIs with senior clinicians. Participants were purposively sampled from each health facility in consultation with the facility in-charge.

**Fig 1:**
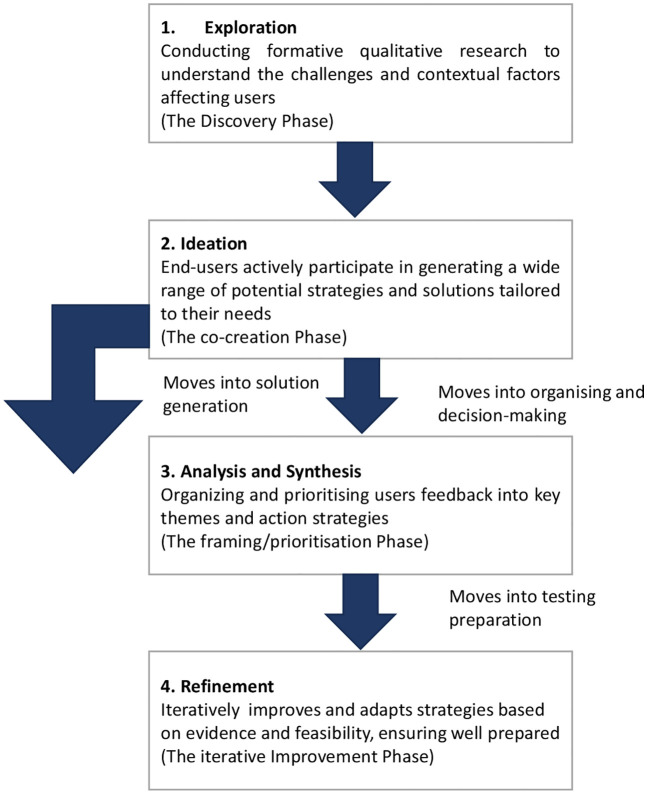
Summary of human-centered design (HCD)-inspired methodology used in the TASKPEN study adapted from Harte et al, [21].

We applied a four-stage abridged adaptation of HCD to structure the co-design process (Fig 1). The Exploration phase involved conducting formative qualitative research to understand contextual challenges and user needs related to HIV/NCD integration. During the Ideation phase, intervention deliverers participated in generating a wide range of potential strategies and solutions tailored to their needs. The Analysis and Synthesis phase focused on organising and prioritizing user feedback into key themes and actionable strategies. Finally, the Refinement phase iteratively improved and adapted the strategies based on evidence and feasibility, ensuring they were well prepared for pilot testing.

### Data collection: Co-design workshops

Co-design workshops were conducted once in each facility over a series of three-hour meetings held within a week, with a total of 75 participants and an average of 15–20 participants per workshop. We used maximum purposive sampling to recruit workshop participants across the four facilities, ensuring representation across professional roles, experience level, gender, and clinical responsibilities relevant to HIV/NCD integration. Participants included nurses, lay healthcare workers, clinical officers, pharmacists, laboratory technicians, and lay/community health workers, who receive training to deliver healthcare services and bridge facilities and communities. This diversity ensured that multiple perspectives were considered and cadres were well represented, and gender balance was maintained across the workshops. We included healthcare workers directly involved in HIV and/or NCD care for at least one year to ensure operational knowledge of HIV/NCD service delivery, challenges, and facility–community interfaces.

For each workshop, a participatory, iterative design process was used with one intervention design team at each site, composed of four members of the research team and frontline health workers who would be primary deliverers of the intervention. An initial agenda, developed by the research team based on findings from the formative Exploration phase, guided the discussion, and the meetings were intentionally collaborative and interactive. Health leaders introduced the workshops, and participants were encouraged to be open and creative during discussions; health workers formed teams based on their involvement in HIV and NCD management. Two facilitators led the HCD process: the first author (a doctoral student/research fellow) led the workshops, supported by research assistants PFS and PC and the project study nurse PS. The study team took detailed notes during each session, reviewed outputs and notes daily, and dialogued to identify key questions, emergent insights, and direct feedback on the TASKPEN intervention. A summary of specific activities used in the co-design workshops are described in Table 2.

**Table 2:** Summary table of HCD Activities to Support the Co-design of Intervention sub-components.

Phase	Participants	Main Activities	Contribution to Adapting Implementation Strategy Components
Formative Qualitative Data Collection (**Exploration Phase**)	- 4 FGDs: NPHWs (32 participants) - 4 FGDs: CHWs (32 participants) - 20 IDIs: PLHIV with NCD co-morbidities - 6 KIIs: physicians	- Semi-structured interviews (KIIs, IDIs, FGDs) using guides adapted from CFIR domains - Onsite observations of routine services	Generated foundational insights on barriers, facilitators, user needs, and health system context to inform intervention design
Co-Design Workshops (**Ideation Phase**)	- 75 frontline HCWs across 4 facilities (nurses, lay health workers, clinical officers, pharmacists, laboratory technicians) - Design team: doctoral student, research assistants, project nurse	- Presented formative findings visually to participants - Facilitated participant reflection and feedback - Conducted brainstorming and idea generation using “ How Might We” questions - Documented participant ideas in field notes	Generated novel implementation sub-components through collaborative ideation with end-users; identified potential barriers and facilitators to intervention delivery
Thematic Analysis & Feedback Workshop (**Synthesis Phase**)	- Design team: doctoral student, research assistants, project nurse - TASKPEN research team: investigators, study managers, research fellows, study clinicians	- Transcribed and organized workshop recordings into Microsoft Word documents - Conducted rapid four-step thematic analysis using summary tables, matrices, and affinity maps - Organised and prioritized feedback into themes and candidate strategies - Compared outputs across facilities and reconciled variations by integrating complementary ideas and discarding infeasible or duplicate strategies	Synthesised and prioritised feedback into actionable themes and recommendations; produced evidence-informed prototype recommendations aligned with HCD principles and formative findings
Prototyping & Refinement Meeting (**Refinement Phase**)	- Design team: doctoral student, research assistants, project nurse - TASKPEN research team: senior investigators, study clinical team, study managers	- Presented synthesised findings and prototype ideas to TASKPEN team for validation - Conducted feasibility and resource assessment - Evaluated all ideas against three criteria: pros, cons, and feasibility	Validated prototype recommendations; refined and prioritised 4 core sub-components of the TASKPEN implementation strategy; ensured intervention refinements remained grounded in participant feedback and HCD principles
Protocol Adaptation	- Senior study investigators	- Incorporated identified sub-components into the TASKPEN protocol during formal meetings - Documented changes grounded in evidence from formative work and HCD process	Enhanced intervention robustness and implementation fit; ensured the strategy aligned with real-world implementation needs and study objectives

Workshops were chosen because their focused, time-bound nature supports rapid generation and evaluation of multiple ideas in a short period. Visual presentations helped participants understand complex problems and communicate ideas effectively. The workshops were also particularly useful for generating a wide variety of solutions through creative brainstorming. Healthcare providers and the research team worked together across the four study sites to refine the proposed TASKPEN intervention. Through these discussions, the team aimed to identify implementation sub-component strategies that could identify, prioritise, and address barriers to usability and improve the TASKPEN package’s utility, uptake, sustainability, and effectiveness.

During the **co-design workshops** at each facility, the design team members reviewed the qualitative data gathered in the exploration phase and shared their insights, experiences, and opinions to get feedback from workshop participants through a visual representation of findings. After the presentation, workshop participants were given time to reflect on the results presented and provide feedback. Questions were then asked about how to implement the intervention better. The following ``How Might We“questions according to Mortensen [22] were used to guide the workshop:

- How might we integrate the TASKPEN package into ART clinic workflows so as not to overburden health workers and make patients wait too long in facilities?
- How might we encourage treatment supporters/lay health workers to take on the extra tasks of educating PLHIV on NCDs and tracing patients who miss an NCD medication collection?
- How might we encourage a workplace environment that embraces HIV/NCD integration such that the TASKPEN package is accessible, desirable, and usable for facility staff?

These questions motivated the discussions that led to the creation of additional and refined strategies for the TASKPEN intervention. Design team activities targeted three core phases of HCD; brainstorming, conceptualization, and creation and included an iteration meeting among the research team to refine the intervention.

### Data analysis

Workshop discussions were recorded and transcribed into Microsoft Word documents, we conducted a rapid four-step thematic analysis using summary tables, matrices, and affinity maps to systematically identify patterns and key themes across the data [23]. A feedback meeting was convened with the TASKPEN research team, comprising investigators, study managers, research assistants, research fellows, and study clinicians. During this meeting, led by the design team, the analysed findings were presented and synthesized through a HCD lens. Insights were visually mapped and organised into themes reflecting potential barriers, facilitators, and unmet needs, directly informing subsequent intervention adaptations and refinements. This meeting aimed to evaluate all concepts generated during the design process.

We systematically assessed each idea against three criteria: identified pros, cons, and feasibility. This rigorous evaluation ensured that intervention refinements remained grounded in participant feedback and HCD principles. The co-design workshops generated ideas, explored concepts, and facilitated brainstorming sessions with intervention providers to create a shared vision for HIV/NCD services. This prototyping meeting focused instead on collaboratively validating and refining those ideas. Specifically, the meeting gathered structured feedback from the research team, identified usability challenges, and validated assumptions made during earlier design phases.

In the Protocol Adaptation, senior investigators formally incorporated the co-designed implementation sub-components into the TASKPEN study protocol. Drawing on evidence from the formative qualitative work and the preceding HCD phases, the team systematically reviewed, refined, and documented each sub-component to ensure coherence with the trial objectives, clinical workflows, and existing health system structures. This process enhanced the robustness and contextual fit of the intervention by aligning the integrated HIV/NCD strategy with real-world resource constraints, stakeholder priorities, and implementation feasibility in participating facilities.

## Results

### HCD workshops participant characteristics

Seventy-five healthcare workers participated in the workshops across four health facilities, representing a diverse range of roles within the healthcare system (Table 3). The largest group consisted of 30 nurses, with an average age of 35 years and an even gender split of 15 males and 15 females. Nurses had an average of eight years of experience and typically held either a Diploma or Bachelor’s degree in Nursing. Lay health workers formed the second largest group, with 27 participants. They were slightly older on average at 40 years and predominantly female (74.1%, n/N = 20/27). These workers had the least professional experience, averaging eight years, and typically held a psychosocial counselling certificate. The workshops also included eight clinical officers, this group was slightly male-dominated (62.5%, n/N = 5/8). Clinical officers had a Diploma in clinical medicine. Six pharmacists participated, with an average age of 38 years and an even gender split. They had an average of 10 years of experience and held Bachelor’s degrees in Pharmacy. The smallest group comprised four laboratory technicians, with an average age of 36 years and an even gender distribution. They averaged seven years of experience and typically held a Diploma in Laboratory Sciences.

**Table 3:** Characteristics of HCD workshop attendees.

Participant Type	Number	Average Age	Gender Composition		Average Years of Experience	Highest Education Level
			Male	Female		
Nurses	30	35	15	15	8	Diploma/Bachelor in Nursing
Lay Health Workers	27	40	7	20	5	Certificate in psychosocial counselling
Clinical Officers	8	42	5	3	12	Diploma in Clinical Medicine
Pharmacists	6	38	3	3	10	Bachelor in Pharmacy
Laboratory Technicians	4	36	2	2	7	Diploma in Laboratory Sciences
Total	75	–	32	43	–	–

### Integration champion

In response to concerns about sustainability and ART clinic ownership of HIV/NCD integration, a champion was proposed to be selected from among the providers. This champion would be responsible for spearheading various activities at the facility, including sharing information about integration activities in the ART clinic and other relevant departments, and communicating with these departments. Participants also suggested expanding this model, appointing focal persons at the sub-district or district level to supervise and sustain integration efforts across facilities.

**Refinement and Final Strategy:** The refinement stage consolidated these suggestions into a single, coherent, and sustainable role: an HIV/NCD integration champion at each facility/ART clinic. This dedicated healthcare worker would be responsible for providing onsite leadership, coordinating integration activities, supporting clinical teams, facilitating interdepartmental communication, and leading data reporting efforts during implementation. This decision ensured the role was embedded within sites, mirroring the successful, clinic-focused champion model used in HIV programs.

### Weekly NCD medication bulletin

To address the challenge of inconsistent supply of NCD medications, providers recommended introducing a weekly bulletin listing available NCD medications to help them identify which NCD medications were accessible at the pharmacy. The pharmacist would share this list with all providers, allowing them to prescribe second-line medications if some first-line NCD drugs are out of stock. This bulletin would provide up-to-date information, enabling providers to prescribe available first- or second-line NCD medications, thereby preventing unfulfilled prescriptions, minimising treatment delays, and fostering more efficient inventory management.

**Final Strategy**: Circulating a weekly facility NCD medication bulletin by the pharmacist was added to improve the visibility of medication stock for the existing supply chain strengthening efforts.

### Dashboard reporting of HIV/NCD cases

Dashboard reporting, a visual representation of key performance indicators was proposed to provide an at-a-glance overview of NCD screening and case identification across departments. This would empower providers to use data for quality improvement and decision-making.

**Final Strategy**: Dashboard reporting on the number of PLHIV screened for NCDs and the number of NCD cases identified was integrated to enhance existing data review processes by adding HIV/NCD indicators.

### Community sensitisation

Participants noted that CHWs had been instrumental in promoting HIV services and could play a similar role for NCDs. By sensitising the community and providing clear information, CHWs could help overcome barriers related to stigma, low health literacy, and lack of awareness. Participants emphasised that when clients are aware of the full range of services available, they are more likely to seek care proactively and hold providers accountable for comprehensive management. By educating clients about which conditions can be managed within specific departments, community sensitisation fosters patient awareness.

**Final Strategy**: Community sensitisation on the TASKPEN package was adopted to supplement clinic-centered care by fostering demand and accountability at the population level.

### Final implementation package

**Fig 2:**
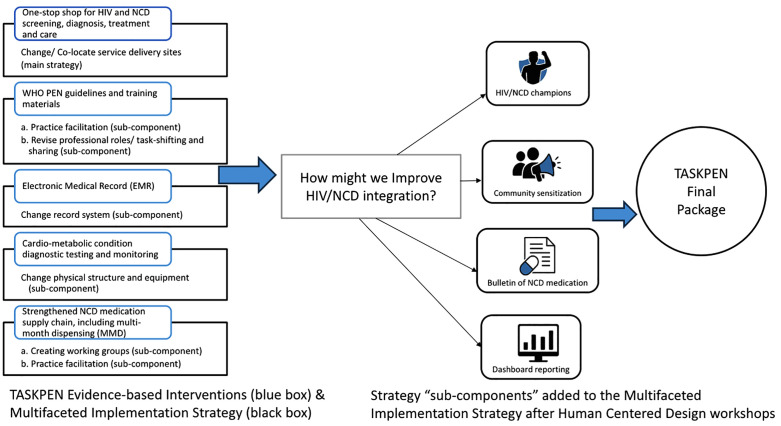
Summary of the multi-faceted implementation strategy initially developed as part of the TASKPEN package and those sub-component strategies introduced through the HCD process.

The original TASKPEN package included a multifaceted implementation strategy centred on HIV/NCD service site co-location as the main sub-components, with sub-component strategies of task-shifting and sharing, practice facilitation, changing physical structures and equipment, changing the record system, and creating working groups. In response to challenges identified during the HCD workshops, additional strategy sub-components were introduced. These strategy sub-components included HIV/NCD champions, medication bulletin, dashboard reporting, and community sensitisation. Fig 2 below presents the original TASKPEN package consisting of five evidence-based interventions and their associated multi-faceted implementation strategy components. The subsequent application of HCD methods aimed to identify, refine, and integrate additional sub-components to address context-specific barriers and enhance feasibility and acceptability. Evidence informing strategy selection came from (1) previously published integration models, (2) national HIV/NCD guidelines, and (3) facility-level insights from the exploration phase.

The four sub-components, HIV/NCD champions, weekly NCD medication bulletins, dashboard reporting, and community sensitisation, were explicitly integrated within the existing multi-faceted implementation framework as complementary enhancements rather than replacements (Table 4). For example, the champions augment task-shifting and practice facilitation efforts by providing on-site leadership, while the bulletin improves visibility of medication stock linked to supply chain strengthening efforts. Dashboard reporting enhances existing data review processes by adding HIV/NCD indicators. Community sensitisation complements clinic-centred care by fostering demand and accountability at the population level. Operationalising these sub-components requires additional inputs, including training for champions, production and dissemination resources for bulletins, IT support for dashboards, and engagement with community health workers and local leaders for sensitisation campaigns. These requirements were acknowledged in the implementation plan to ensure adequate resourcing and sustainability.

**Table 4:** Refined Synthesis of HCD insights for integrated Care implementation.

Strategy (HCD Sub-Component Idea)	Insight leading to generation of sub-component	Implementation Focus	Outcome Targeted
HIV/NCD Champions	Lack of coordination, continuity, and accountability across departments.	Defining dedicated personnel roles ensures integration activities are embedded in routine care and supported by high-level oversight, addressing the structural silo problem.	• Acceptability • Feasibility • Fidelity • Sustainability
Weekly NCD Medication Bulletin	Patient frustration and delays caused by frequent NCD drug stockouts and resulting inappropriate prescribing of unavailable drugs.	Provides real-time, high-fidelity information to prescribers, allowing immediate identification of alternatives and reducing patient and HCW burden.	• Efficiency • Error Reduction • User Burden • Contextual appropriateness
Dashboard Reporting	Fragmentation of patient data (NCD screening separate from weekly HIV/ART data) and lack of a holistic, visual overview of client status for timely action.	Streamlining data capture and visualisation to support clinical decision-making across integrated service points, reducing time constraints and cognitive load for busy staff.	• Usability • Workflow Integration • Low Cognitive Load • Satisfaction
Community Sensitisation	Client awareness regarding the availability of comprehensive integrated services (HIV/NCD) and confusion regarding referral pathways.	Fosters patient awareness and creates community demand, thereby maximising uptake (adoption) of available integrated services and fostering health-seeking behaviour.	• Engagement • Appropriateness • Adoption • Penetration

## Discussion

This paper describes how HCD was used to adapt evidence-based solutions for integrating HIV/NCD services through engagement with intervention deliverers, and to share insights on specific suggestions generated from the HCD process. We describe the suggestions made by healthcare providers during HCD design workshops to improve patient care, reduce referrals, and improve the availability of NCD medications and diagnostic testing. By integrating HCD principles to refine the TASKPEN subcomponents, an HCD approach fosters empathy during the exploration phase as indicated by Kang et al, [24]. This depth of empathy also helped us make decisions that would achieve better outcomes and enabled rapid analysis of prototypes emerging from HCD methods. The HCD process refined the TASKPEN multifaceted strategy, which included an HIV/NCD champion, a weekly NCD medication bulletin, dashboard reporting of HIV/NCD cases, and community sensitisation. This process helped to improve the adoption, appropriateness, and feasibility of HIV/NCD service integration within routine HIV care.

The iterative cycles of the design process allowed for the identification of strategies before trial implementation, thereby enhancing the intervention’s potential impact on the study outcomes. The four core suggestions from the design team focused primarily on incorporating implementation strategy subcomponents to support the introduction of the TASKPEN intervention and on enhancing provider and patient uptake. The TASKPEN intervention combined evidence-based practices and a multifaceted implementation strategy to improve the management of NCDs among PLHIV [17]. The participatory HCD approach involved healthcare providers as intervention deliverers, leveraging their experiential knowledge to foster ownership and tailor solutions to each facility’s context. Such involvement was expected to enhance intervention uptake and sustainability, consistent with findings from other HCD applications in complex health systems. By involving healthcare providers early in the intervention design process, we addressed their concerns and built essential partnerships with local stakeholders, both within and outside the facilities. These partnerships proved crucial for subsequent TASKPEN intervention piloting, as noted by Leung et al, [25]. The HCD process yielded valuable insights that significantly shaped our intervention and its multi-faceted implementation strategy.

The four co-created suggestions, HIV/NCD Champions, the Weekly Bulletin of NCD medication, Dashboard Reporting, and Community Sensitisation, were direct solutions to barriers identified by the healthcare workers during the formative exploratory phase. The suggestion for an HIV/NCD Champion, for instance, was driven by the perceived need for defined accountability and coordination across fragmented facility departments. Participants viewed this role as crucial for embedding new practices into routine care and promoting long-term sustainability. The suggestion for a Weekly NCD Medication Bulletin was a direct response to the supply chain’s operational challenges, which frequently leads to drug stockouts. Providers identified the need for real-time information, not just about stock levels, but also about available second-line alternatives, to enable them prescribe what is available and manage clients effectively without causing delays. This reduces the user burden on clinicians facing resource constraints. The integration of NCD data into Dashboard Reporting addressed the fragmentation of patient data across multiple touchpoints. HCWs emphasised the need for a unified, visual overview of patient status to streamline reporting and clinical decision-making. Finally, Community Sensitisation was identified as vital for patient engagement and adoption. Workshop participants highlighted that informed clients, educated by community health workers, would not only be more likely to seek integrated care but would also be empowered to remind HCWs to provide holistic management, fostering shared accountability and coordination across services.

Despite these promising components, challenges remain. Stockouts of NCD medications are frequent in Zambia due to supply chain limitations, which may limit the effect of weekly bulletins unless accompanied by system-strengthening efforts. Community sensitisation, while resource-intensive, is seen as vital for demand generation and stigma reduction, with effectiveness dependent on culturally tailored, context-specific strategies. HCD methods led to the identification of contextual problems and suggested strategies for mitigation that would have been ignored were it not for the HCD process.

### Study limitations

While we did not include key stakeholders such as hospital management teams, policymakers, and PLHIV with NCDs as HCD workshop participants, we did capture their perspectives through concurrent formative qualitative research with these groups. We shared their insights during the HCD workshops. However, having them directly participate could have provided additional valuable insights and potentially enhanced the intervention’s feasibility and sustainability. Future iterations or scale-up of similar interventions should prioritise engaging these groups during HCD activities to improve the intervention’s acceptability, effectiveness, and potential for successful implementation within the broader health system. Second, the duration of the workshops may have been insufficient to fully ideate, analyse, and synthesise solutions. However, we did not prematurely cut off any dialogue and had to balance the need to complete the workshop with the limitations imposed by busy healthcare workers’ work schedules. Finally, while we applied the Consolidated Framework for Implementation Research (CFIR) [20] in our formative research, we could have more explicitly mapped HCD workshop recommendations to CFIR domains and constructs.

It is also important to note that HCD processes often involve extensive research, iterative testing, and close collaboration with intervention deliverers. This can lead to longer project timelines than traditional design methods, which might be impractical for time-sensitive projects and thus create antagonism among stakeholders involved in project development and implementation. Additionally, conducting user research, prototyping, and testing requires significant resources, including financial, personnel, and technology support. Small budgets or limited access to specialised tools can hinder the effective implementation of HCD. Lastly, the absence of a formal evaluation of the HCD process itself, noting that participants’ statements about value remain informal and qualitative rather than quantitative. Future work should incorporate structured process evaluations to assess the influence of HCD on implementation outcomes rigorously.

## Conclusion

From our findings, applying HCD helped adapt evidence-based interventions for HIV/NCD service integration. Our HCD workshops identified solutions to challenges providers and patients may face in delivering integrated HIV/NCD services. The HCD approach involves understanding the context and empathising with end users, ideating potential solutions, prototyping and testing them, and iterating based on participant feedback. HCD design should be considered in implementation science studies in LMIC settings to refine implementation strategies that overcome challenges to the delivery of evidence-based practice in healthcare and motivate providers and patients to sustain the needed behaviour change. When stakeholders such as healthcare providers are involved in identifying solutions to real-world challenges, they are more likely to propose and participate in strategies that are sustained. Stakeholders, therefore, have an essential role to play in the adaptation of evidence-based interventions for integrating HIV and NCD services. In healthcare, HCD offers the opportunity to develop innovative, user-driven solutions to complex problems impacting clinical and public health outcomes.

## Supporting information

S1 File HCD Workshop 1. (DOCX)

S2 File HCD Workshop 2. (DOCX)

S3 File HCD Workshop 3. (DOCX)

S4 File HCD Workshop 4. (DOCX)
