# Supplementary material for: Applying human-centered design to adapt a multifaceted implementation strategy for integrating HIV and NCD services in Lusaka, Zambia: Healthcare worker perspectives
Source: PLOS Glob Public Health. 2026 Feb 2;6(2):e0005879. doi: 10.1371/journal.pgph.0005879 (PMC12863476; doi:10.1371/journal.pgph.0005879)
Supplement: S1 File — (DOCX) [file pgph.0005879.s001.docx]

**Interview Date: 28^th^ January 2022**

**No of participants:**

**Site: Chawama level on hospital**

**Interviewee category:**

**Interviewer: Tulani F.L Matenga**

**Transcriptionist: Tulani F.L Matenga**

**Time:**

**I: so we want to hear your voices, last time we were here I think we were the only ones who were talking right, yes. First of all, if we comment, are we, I know the sister in charge has already said yes, but we want to hear a bit more in terms of what makes you say that these results are true representation of what we discussed here and if there are any concerns that you have or maybe I should begin by asking a question sister Penda.**

**I: Yah whichever way**

**I: And I think I want to ask a first question. How might we integrate task and package into ART clinic work flows to not overburden health care workers and make patients wait too long? so two way questions, how do we not overburden your work with this particular task and how do we ensure that recipient of care don’t wait for a long time? Because remember as a study team we are here to provide evidence for ministry of health if it does work this will become the norm in our country to say this is how we are going to provide care for people at the end of the day. So we want to hear really how can we not overburden you as providers or maybe or maybe you are not overburdened?**

R: laughs.

**I: Yes.**

R: first before we answer that question, so for hypertension is very easy for us to not, now on diabetes are we going to be checking every client that comes for ART, are going to be doing random bloody Sugars or passing blood sugars and urinises, okay urinises we also do. So on the diabetes part that’s where I want to find out it even be very easy to answer that question.

**I: So this all idea of this study is all about to see if this is something which is workable. So as we come on ground in the two facilities where we are going to do the pilot, we’ve provided some glucometer during the course of next week we are doing a survey, we want to check at random when people walk in, so we’ve provided the glucometer because those are things we need for us to see, is true that there is some existing of diabetes in our general community under the ART team, so that’s one thing yes. We will provide those glucometers so that we check for those, hypertension of course reading machines will be available. We will do the measuring we are going to orient people who are going to be helping us with measuring east circumference as well as the west circumference because that’s where we have prominent part disposition, then we will be also doing some labs, we will be getting some labs where we check for confirmation of fat levels in the blood stream so to say. So we are going to do that we will be provided with point of care, where people like me the nurses will be able to operate not really like in the lab because in the lab there are other lot of things that are happening. So that point of care where by if I need to do this person is okay then we find the bloody sure is high then circumference is over the normal so to say the normal stages, then we will be able to do that, to perform the test and also have the result because it will help me or guide me to make right decision yeah, so basically that will be done.**

R: Okay, then if

**I: You wanted to say something?**

R: I wanted to ask another question, are also just focusing on diabetes and hypertension unlike common things, because there are also other things like mental disorder. How far is your integration of NCD with ART?

**I: Thank you so much, so for this study we are focussing on three conditions, Hypertension Diabetes and (unclear speech). The lesson learnt from these conditions, this is about 9 to 12 months study, the lesson learnt from here will be applicable to these other non-communicable disease like mental health.**

**I: Okay, any other, you can comment as well, you can give us idea we have come to people on the ground, us on the ground we know how best it can work, because if it tells us to say, just call them and check their blood sugar, us we know that we need first to talk to them the importance isn’t? Even if we have just come to say just check their blood sugars all of them who have come but you know that it will not be easy to just tell everybody who have come, come let’s check sugar, it will not be that easy so,. We will give you the idea to say we don’t just call them come, we first need to sensitize them the importance of knowing their blood sugar and how it is going to help them in the future so now you will consolidate on that. That’s why we have come to you the people on ground, you know better, we know our community very well, we know what is working better. So most of the times what limits us is resources so once we have those ideas we can put them together then we will be able after have the results we will able now to source for resources so that we are able to help our community.**

R: I don’t think we will be burdened in the sense that provided at triage we are doing even diabetes can be one of the things other than the BP, we can also be doing random blood sugar, and them the point of care I don’t know where we are going to put it if it will be the person working from (unclear speech) will still do it as part of the routine work they are doing, for me I feel we will not be overburdened unless sister hope or sister violet think different.

**I: Sister hope, where are you (laughs).**

R: for us nurses, that’s what we do at triage when we come, when patient come, so most of the sometimes hypertension clients we usually do it from triage, so adding the diabetes it not an issue just that it will be taking a lot of time for the clients because it will non-communicable disease (NCD) as well as ART.

**I: So then how do we reduce the time that recipients spend, how do we work around the issues of time remember we are trying our best so that this client is spending as little time as possible that’s why when we start you will be seeing us observing the only thing we will be interested is, how much time they are going to spend the time they walk to meet you, time that they leave, that’s also our interest. So how do we reduce this time that this particular recipient of care is spending here at the facility?**

R: I think from triage part like, we are maybe like two 3 nurses working at triage so that someone is doing the, checking the sugar and other things so that we can be fast, we have 3 clinicians there.

**I: Okay, so to have more providers at certain point right, okay.**

R: So can we say that, we strengthen the screening aspect of these non-communicable disease so that at least even when we do past triage we know which one is on a particular need, I think we need the screening criteria (unclear speech) maybe if we imagine a point that we do not have other partners, maybe from that screening point we assess how many put a burden on already available staffs then we assess how much support will be needed in terms of human resource and maybe materials.

**I: Okay, Strengthening what is happening the screening at triad right? So that we are able to ahmm now capture exactly how much human resource do we need and things like alright, people are talking now, so sister you see, you have opened up when the boss speaks everybody else speaks**

**I: It is automatic you see?**

**I: Yes (laughs)**

R: For me I was thinking it could really be helping if we could know the numbers like how many clients have NCDs, if we have the number you would know if we could meet most of or not. Because if we have a lot of people that have, I don’t know [/a lot/] if clients with NCDs we would, I feel there will be need to have a separate clinician that will be attending to these clients who have both ART and NCDs. So that obviously that will lessen work for both the clinician and also working on the, looking at the hour of the client spending at the facility.

**I: Alright okay, so the first questions, by clinicians you mean the Doctors or Clinical officers?**

R: Both

**I: (cross talk) The clinical officers both, okay alright? So remember when I said we are task sharing and task shifting, meaning we are not relying on this particular cadres alone but even you, you will be able to manage conditions, you will have access to SMARTCARE pops up and say this particular individual also has an NCD and needs this particular medication, so you are managing for both, remember you have shared the task. So we are not relying only on 2, or maybe a Doctor yourself and a clinical officer but the task has come down all the way to the treatment supporters. So which means the treatment supports have got a role to play in there as well, so I don’t know if I have answered something, or I have left out something as well?**

R: No you have answered.

**I: Okay alright, any other? Or maybe I should ask, or maybe you want to ask?**

R: When you look at it, in that aspect, in terms of just amm, screening, I mean can I say (unclear speech) maybe medical examination of this client, on that practice, I am also looking at the pharmacist. On the part of the pharmacy it is not everyone who can just go the pharmacy and prescribe the drugs for DM patient and for now. We shouldn’t just look at it, in terms of the clinician and also we are supposed to look at it in terms of the whole management of a client in terms of walking and walking out.

**I: Uhm**

R: Also infrastructure, it should also be looked at.

**I: That is very important right, infrastructure itself, being able to, to accommodate as many people, so I like what you have said, like a holistic approach where different people are playing a different role. I think that is also a part that we mentioned that we hope that everything could happen within the ART, this person should go around Zambia from the ART clinic, they go to OPD by the time you are finishing.**

R: It is 19:00

**I: Even the bread you ate in the morning is done, isn’t it, so that is our hope that this one come and at least within an hour everything happens and I can go home, I can go and work for the Indians in Kamwala, I can go and work in the wholesale, right here in town so that is what we are basically trying to achieve at the end of the day. Any other comment, so with what I am getting here, is that we are not going to overburden you right? I am only seeing, I am only hearing a few.**

**I: Sister hope, you are our only hope here.**

R: (Group Laugh)

**I: We are not going to overburden you.**

**I: You are our only hope (chuckles) with this line of though, is there, has it come with a very big load to overtake us?**

R: Uhmm, I can say no, because we are here to help the clients, cause moving from ART, the clients goes to OPD most of the time it is packed and everything, so it is a good initiative, I am sure it will not overburden us, we are not carrying a burden at a goal like.

**I: I see, okay, okay. Alright thank you very much.**

**I: Throw the ball to another person first**

**I: Oh yah the ball.**

**I: Because they are the ones, sister. (Group laugh)**

R: Yes the clinicians

**I: (Laughs) Yey so we want to hear from the clinicians maybe you are going to say ahmm, us we are trained to do this now we start sharing the responsibility with other why?**

**I: It is our job, why should they grab it.**

**I: This is our job, why should they grab (cross talk) oh maybe you are happy to say one less responsibility for me. Yah we want to hear from you as well**

R: Yah, first of all, I am happy about this idea ahm, and I feel it will not overburden us because all along this is what we have been trying to do, I would say, we have always been doing it, only that we have got nothing, the resources, the drugs to use, the resources. So I feel it is something that we have always been doing but if we receive support it will help us and then it is a good thing to share responsibilities. We always intend that everyone in the department knows what they are doing and ahmmm, if ahm one that is supposed to do it is not there every other person that is available should be able to carry it on, so I think, it is good.

**I: Okay, you had something to say.**

R: Yah I think with the idea of putting SOPs this will be really helpful, and you know even if, imagine, I will give you an example, ahmm, in a way we have been working task shifting has already happened and when they are, when we have SOPs it will also increase and learn more. You have a client who has high BP, if there will be a client waiting, but there is a client who will enter in your office, but when they come the nurse if already there to give medication, this medication you know where to start from. So it will be easier for us to interact, I think like he said, something that we have been doing, we must be just honest that for DM maybe with essdimia we have not been doing much because we are really limited in terms of maybe glucometers and everything. And I just have, the fear that I have is that, you know for ART, we are a lot of people on medication. Like here we have like almost 12,000 people that need to be screened for all the 3 diseases, maybe when starting the screening, maybe it will be difficult we have a lot of people to do at the same time. But when we streamline them, we have just a few maybe they continue it will be easier for us.

**I: That is a very good point, this system that we are trying, because we are just trying to blend into what is already existing, I know with ART, we have the 6 MMD isn’t it? Multi-med-dispernsion so people are not over, we are not having congestion in the facility for now, we are seeing by 12:00 at the most, by 12:00 the crowd has already gone we are seeing the once off clients right. And people now have got even that opportunity of even interacting with us, I think me I will only have time around 12:30 and we are agree with them and give them that appointment, so we just want to come in the already existing system. So even as we bring in this idea, it is not about calling the market to say, all of you come, as they come, then we are also doing these other things, we are getting to know, who is diabetic who has dyslipidemia, like high cholesterol levels who is really hypertensive and then we keep on monitoring them like that and then also giving them appointments. We want also to reach a stage where by hypertensive clients they are given 6 MMD, diabetic clients they are seen twice in a year or 4 times in a year, every quarter, that’s the point we want to reach, that’s the goal we want to reach and also they have availability of their supplies of medication, that’s the goal. The whole study is about that, so really, that’s the idea behind it, what is already existing, if I come for DSD, within DSD let also by BP be seen within DSD if they disperse for me DSD, they disperse for me 6 months, at least, even 3 months for my BP and then I will be home and dry, I will be be doing other jobs I am supposed to do, that’s the whole idea. Not really like okay now, let us call all of them yah they can be campaigns there can be campaigns under ART right, to say we have got a VL campaign those who don’t have VLs. Yah we can have such, once of things happening, but eventually all in all, we want it just to blend into what is already existing.**

**I: Any comment?**

R: The comment was on medication as well, I know you come with the glucometer and everything what you have said, it is very true, our clients really suffer, you find that you see them today, you give them 6 MMD, but ahm, you give a prescription for (unclear speech) maybe for 2 weeks and ahm they might not come again, then our clients take ART to be the most important they will not come. So is it any, are we going to have other support in terms of medication, because if you support with a glucometer are you having a way of supporting with medication.

**I: Yah okay, thank you so much, that is a very valid point because we cannot diagnose and tell them, you look like your sugar is beginning [/rising/] and then we end there. Then we will worry this lady a lot, but they told me I feel dizzy, they said it is sugar and they will die faster. So uhmm, this discussion as we have come to the facility, it started way back engaging stakeholders, ministry of health, all those consultants at that level. Hence the pharmacy component is not something which has been overlooked, we have engaged the pharmacy people and I am sure people who are in the pharmacy they have already interacted with the pharmacy person from our end whose been on ground to check what is there, what is not there and everything. And to that we have worked on strengthening the pharmaceutical aspect of it, as far as from the ministry level. I know we have ahm, we have politics behind it, where we would say we order drugs from MSL, we receive there are no drugs, they are out of stock, but when it goes out there, Eva says we have drugs in stock, there are drugs in stock. So we started from that level we are strengthening the system where ministry has made an agreement and have made a promise to say they are going to improve on the NCD drug acquisition so that we should have that available. But besides that as a project we are contributing a small percentage to backup in case ministry really or takes long, their procurement takes long and we will not let out clients to suffer after we have diagnosed them, then they start just coming without any drugs. So we have a small ahmm, percentage just as a backup to what ministry but we haven’t ended there, we will be still pushing. It is our pharmacy technical group who are still working with them and ensuring that really the drugs should be available. And hence as of today you are talking of, you are talking with ahmm, my colleague we came, we have been reassured by ministry that we have procured a good number of, at least not less than a month stock of drugs for NCDs.**

**I: Alright, thank you very much sister Penda, any comment, do we have people from pharmacy? (Pause) We don’t have any pharmacy people, okay your comment from pharmacy? Ahm, the boss says that pharmacy chishimba**

R: Chishimba

**I: Chishimba yes, so it is the heart for what we are trying to do, we cant take and leave people, we need to provide the drugs, so it is a very important, one of the biggest components of our study, so we need to hear some comments from you, in terms of recipients of care and also getting their drugs also when they come. Holistic approach, cause we want, treatment and drugs in one visit then I go home and I come back after 6 months for example.**

R: Ahmm, I am just hoping like she said that the medication will be available, that there will be that supply of drugs, I am just hoping it will really put into consideration cause even as at now they are even, at OPD they are even unable to give a month [/large/] of medications out there. Even ordinary paracetamol sometimes we don’t have in stock, I am just hoping they will really put in so much, considering that they said that, considering this same procedures that we want to put in place, I am hoping, they will step in, maybe things will change.

**I: Okay, alright.**

**I: And what will be your role? We want you to you have told us what you are hoping for?**

R: So obviously for us, we need to ensure we order enough drugs, we know the consumption of each particular drug and to ensure all the drugs are available and through reports sometimes to avoid stock out.

**I: Okay, thank you, so do we have the treatment supporters or community health care workers, do we have? Yah, there anyone else? So now I want to hear from you, because you are the team that is really interacting with, because of some of these challenges, some of our recipients of care are coming to you, they become family and they become known to us and as treatment supporters we have also this relationship with them. So I am trying to find our in terms of how can we support you so that you take on this extra task? Because already with HIV you are good, you have these roles that you are playing you are in the community, you are dispensing medication, you are calling them reminders, you are their support system here, you are their friend. You come together, like we heard from some of you, you take them, you hold their hand and remove them from the ART clinic to the OPD, so we want to know how do we support you, so that you are able to take on this extra task? You can start ma’am**

R: As for us treatment supporters, uhm, maybe we need to be trained.

**I: Maybe, you are doubting?**

R: I am not doubting (Laughs)

**I: What do you need, we need to discuss.**

R: We need to be trained.

**I: Any other needs that you have?**

**I: What kind of training do you need? Cause training is broad ey?**

**I: Yah, very true**

**I: We will start teaching you how to eh, which I don’t even know, how to use the ECG (Laughs) okay**

R: How to test, we need to know, then sometimes you find that, when you test, you find that maybe the sugar levels for the patient is high, we need some (unclear speech) on care

**I: Yes my brother there.**

**I: Apart from the training, what do we need to be equipped?**

**I: Support yes.**

R: Mostly training, orientation at least on how to do vitals, that when (Unclear speech) us treatment supporters when we know what is going on with vitals, we will be able to refer that this client needs to go this side, this client need to go that side, so that at least we work on time.

**I: Okay, thank you, so we want to hear from others as well, cause I have heard from the treatment supporters, how do we encourage them, because remember, encouragement will not only come from us as a study team, because we are going to be here for a very short period of time, to collect away evidence present it and if ministry this is good, then again it is going to be your baby. And then again in terms of other carders how can it, you know encourage our treatment supporters to take on this extra responsibility because remember I mentioned some tasks that they think they can do cause they mentioned it and how do we support them to do, some of the things they said they will do for example when it comes to managing people that are living with both conditions? So I haven’t heard, I want to hear from people that I haven’t heard from, I need to hear your voice so that I say that voice is familiar I say hello. Yes my brother there?**

R: I think ehmm, how would we be encouraged, I think training can be okay, so that we have the necessary knowledge about what we are doing.

**I: So our training will also, the good news is that among the team we also have Doctor Telo, I think after we are done with the pilot part, when we come back, to do on a larger scale she is someone you are going to be meeting who is designing ahm the curriculum for training providers, so in terms of training we are going to be here to support you in terms of that as well, but we also want to hear other ways that we can encourage you, from the other providers, how do we encourage them, cause remember we are a team and ART clinic has worked successfully. Zambia is, Zambia is actually one of the leading examples when it comes to the community health care worker system and how they have help ahm, in ensuring that they have success in the ahm ART Clinic. Any suggestions? You have something to say I can see, we want to hear from you. You can’t because you are the people supporting them isn’t it? So I want to hear, how maybe on your part how do you think you can support our treatment supporters, so that they take on this extra task? As an individual what do you think you can do to encourage them? You are still thinking**

**I: How are you going to encourage us as treatment supporters, so that we work even extra hard take up some of your responsibilities while you focus on those much complicated?**

R: In terms of working not to make them feel inferior so to say, lets say they are doing the vitals and things like that they bring up the vitals. Sometimes maybe the person can bring the wrong readings but you are able to teach them to say this is how it is done it is BP, when you see this kind of, its sugar levels you can notify us like that and not making them feel inferior as though they don’t know anything, then we can work together. Make them feel part of the team as well.

**I: Okay, alright, that is very good, mentorship yah, you mentor them, as an individual you are going to mentor someone right? Supporting them closely, individual support right, you had a hand my brother there.**

R: Ahmm, I will start by raising a statement, then I will have a, I will raise a suggestion.

**I: Okay**

R: Usually ahm, in the lab, we receive two containers, one is the total congesstal, restochemistry (Unclear speech). So my suggestion is ahm, for us to ahm, ahm, handle these NCDs, it can be okay to add additional tests such as total cholesterol or liquid profile so that we can yah, some additional test can be.

**I: Alright, the good news**

**I: What additional tests?**

R: Total cholesterol and liquid profile

**I: Okay, yes that’s true, that is part of it, so that point of care machine the aflion is going to be used for that, yah.**

**I: So we have someone who is specialized in lab eh, Chilambwe, so the team is actually made up of people that have been gifted with different things, so we also have that, Chilambwe will be supporting, so once we are on a larger scale, we will be there to support people in the lab and also to ensure that we work together as well. (Pause) ahm so another question that I have for you, call it the 3^rd^ question we know that ahmm, studies come and go at the facility, right? We have seen so many partners who have come here right, they provide things and they go, so what happens most of times is if you had a study maybe providing, what are some of the things that studies provides?**

**I: (Laughs)**

**I: Take for example, cause I was part of a study that was providing HIV testing kits right at the facility, we were at Chipata level one providing testing kits for a good 2 years at MCH. So after the study finished then we packed our bags and that is how we went, so most studies, once they are finished that particular thing they were doing also ceases to exist. But with us what is different is that we are trying to provide evidence to implement, we are saying after we are done creating evidence we present to ministry. So ministry is a, ministry of health is also our partner, you see our logos we have UNZA, we have ministry of health we have CIDRZ cause we are working altogether. When we are done with our couple of years we are going to say us our facilities take over, this is how we envisioned our things to be happening then we step back as well and see where else we can support the health care system, so in short I am trying to ask, how do we sustain this? In our absence how do we ensure that ahmm, NCD/HIV integration will be the norm when we are gone, we will hearing oh when you go to Chawama everything is ART clinic holistically, so I don’t have to move HIV Chawama, NCD Chilenje. So how do we ensure that there is sustainability of this becomes part of your routine services? Because you can hear tomorrow that those for NCDs left, that has stopped, they went back with it to CIDRZ.**

R: I think that can only work if there is consistency, lets say if that side they never run out of the drugs that there are supposed to be and then they have the equipment in terms of testing. Because if today patients come, they are not tested, tomorrow they are not tested, they are told to go back to OPD, within a month if that system continues you just continue to go back to OPD, then there will be an end to that. But I think there should be just consistency in the availability of drugs and the equipment to use in terms of testing.

**I: So what we are saying is if these 2 are happening then we will be able to provide care right? Then we will be able to provide beyond the proposed time of staying within the facility. Any other? Sustainability, yes sister, I haven’t heard your voice.**

R: Actually us clinicians (unclear speech) seeing our clients in a holistic manner, like for example they are sent this side, we don’t send them back to other management we (unclear speech) we also write drugs from ART, we even send also for other investigations. So that would have been good but the only part which can be (unclear speech) if we fail to continue is when we fail to have such supplies. For example, like she said when the machine maybe are done or maybe the supplies are no longer coming this side, then the drugs are no longer been given to us, otherwise we could be doing it, all we need is just maybe sustainability.

**I: Like sister there has said, something has been happening but we need to be consistent with making these particular drug, these particular testing equipment available within the facility, any other? Is there a different language we are speaking somewhere? Or how do we encourage, ensure that our health facility is actually doing this particular approach I think beyond just sustainability itself to go in the future. How do we know that, how do we know that people are being treated holistically, if we say Chawama level one hospital is an integrated hospital. Cause we know some facilities within Lusaka are integrated right? But then we know integration is not happening even though they are claiming to be integrated facilities, so how do we create this environment that enables integrated to happen? Or how do we know or ensure that integration is happening at the end of the day? Apart from we are providing the testing kits, we are providing the medication, how else do we know that it is happening, or how else do we create this environment where this is actually happening and when clients come, they are seen holistically, how do we create this environment? You had a comment?**

R: (Laughs) I never had a comment on that one but I will try, so I think ehmm, of course the partner that is going to be involved in this is the patient so I think when we create that (unclear speech) to the patient, the patient will know that if is go to ART at this, I will get this I will get that. So that itself will help us continue doing the same.

**I: So it’s like uhmm, recipients themselves doing checks and balances to say, they know that they are aware that they to receive integrated care, so they should be able to demand that when they come here right?**

R: Uhum

**I: Okay, great alright, any other ahmm, maybe also to close, I just want to ask now, cause you know these are some of the things that we think. Like I said, me I am not a clinician, I do many other different things and providing care to people is not among them, yah. Ahmm, so these are some of the things that we thought about, when you are not in the system or not on the ground, you can be there to create something in your mind right? And you think this would work, so this TASKPEN is our thinking, so if I say, for example, I gave you time to say, create your own intervention to manage people that are living with both conditions. Because one you know their challenges right? You have been friends with them, you are family now, if someone is, you are seeing someone for the past 10 years, basically they are your family isn’t it?**

R: Yes.

**I: If we say go and develop an intervention or with what we are proposing, what do you think will not work, how would you replace it, to say okay maybe shift, you can say take out number 2 for example, because it wont work at this particular thing, so that is what I want to hear, if you are given the chance. (Pause) if we are sitting together, writing and developing what are some of the ideas that you would bring on the table or how would you reconfigure ahmm this particular approach? So what I am going to do, I think for this question uncle Peter, I am going to say my concluding remarks, I am also going to give you chance to say your concluding remarks sister Penda, then I will throw it now around the room so that all of us can now have a chance to say, me if I was given a chance to manage both conditions. I would create my own building there, where we are saying this building is only for special people living with both conditions right, this is just me thinking, so I am going to go around the room. So I think first of all it’s to thank you for your time we know as health care providers you are really working hard in the times of covid, you have multiple task you are doing but you have created this time to come and sit with us and listen to us. But I am glad that the results that you presented you are saying yes they are true and we are not over representing your thoughts. So for us it is a learning opportunity cause every time we come close to you, we are in a room like this we are learning as much as we can and also when we learn we are able to share and interact, some of us who are teacher we go back and know share feedback with our students and they are aware of what is happening on the ground. So I think those are my closing remarks, to say thank you and also for your interaction with us, hopefully, next time we meet, we will come and present some more findings. So I will ask sister Penda, to also give some concluding remarks, Uncle Peter will say something and then we will go around the room and then the in-charge will.**

**I: (Long pause) Okay so ahmm, thank you very much it has been a wonderful discussion ahmm, we look forward to working with the team on ground and all the contributions which have been put fourth are really valid and they are going to help as well refine the implementation strategy even as we role out to other facilities. We are going to ahm, to ensure that all these contributions will help us to share the intervention so that it is working on ground, so please, please we urge all of you we are a team and for the successful of anything which comes on ground it should have input from the ground.**

R: Yah

**I: So if we don’t have input from the ground, we have our ideas there, they will never work, because the people on ground know best how things can work. So we want what is workable for Chawama as a community, Chawama first level saves the Chawama community, so we want what is best. So even as we come, to the implementation part of the study, we are confident to say really we are going to achieve this and we are going to prove and show ministry of health that really NCDs also they really need their attention in providing all these other logistics as which has happened with the HIV services. I think I cannot say more than that.**

**I: Uncle Peter?**

**I: All I can say is thank you so much for having (Unclear speech) from our team, from the time we came to collect this data was presented, you gave us chance to meet you, we spoke to the recipients of care so this is the feedback. This is a new thing being done on bored and normally when something is being done, there are a lot of misconceptions so we will rely on you and in our team we have got a component of community engagement so we need to give the right messages to the communities. Because I remember way back there was a study we were doing in Matero no it was (unclear speech) and it was index testing so our treatment supporters were going in the field to test the individuals and that was the time there was that issue of gassing, gassing. So unfortunately that day when they saw them with the cooler box, they thought those are the people for gassing, and they were almost beaten to death, so the biggest component is the community. So as we role all this out, we rely on you, to engage the community so that they get the right messages otherwise we very much thankful to you, we will come back to you when we (Unclear speech) thank you.**

**I: Thank you so much uncle peter, uncle peter is a community ahm, community expert, so all things community related he is the one who knows how to go around them. So he is the one to, if you want to go in the community you just run to him and yah, I hope he stands for MP one day ahh (Laughs). For the community but we are still debating which community.**

**I: (Laughs)**

**I: Actually we will bring him to Chawama community, Chawama yah?**

R: He is welcome

**I: Yah so I am going to, when you are talking, please just raise your voice so that we are able to capture your last words, because I think you are very important people here, so I will start with you ma’am**

R: Start at the back

**I: At the back ahmm we picking (Unclear speech) that is the problem when you are teaching and there are people in front you end up picking on the ones in front. So I think we can start from here and then we go in a zig zag manner, my sister you have a (Unclear speech)**

R: (Cross talk)

**I: Yes how would you manage to begin to develop an intervention that needs to provide recipients of care to the people that living with (unclear speech) how would you, what would you add, what would you subtract?**

R: Making sure that the community stuff keep the messages (unclear speech) the glucometers and (unclear speech) record above normal ranges (unclear speech).

**I: Yes ma’am.**

R: This is a very good, (Unclear speech) cause most times our clients die from NCDs and HIV nowadays so I feel it is a very good initiative, then the only thing I would put in place is encourage our clients to come through in a case they run out of medication ensure the same way they stay glued to getting their medications all their ARVs. Like timely management or knowing when they are supposed to come back for their pick ups even NCDs it should be the same.

**I: It should be the same ahm, alright thank you very much, yes. (Pause) No you have to have something to say, you could even make a comment (chuckles) so we will come back as you are thinking about it. So we are not only asking to say what everyone has said, you can comment, you can thank us you can discourage us and say that is not going to work or anything we just want to ensure that we have heard something from you. So we will come back right, yes my brother?**

R: I think it will, I think it will help our client to manage their NCDs rather than them going to OPD just there when they come we manage the (unclear speech) and also we manage the NCD.

**I: Okay, alright thank you very much, you were about to say something?**

R: No that’s all.

**I: That’s all alright my brother there next?**

R: I just want to say, not talking about clients come to collect medicine but I want the health workers (unclear speech)

**I: Okay, anything else? That’s all? Yes ma’am**

R: I would say it is a good initiative, okay, I have worked previously in a center, in a clinic that I integrated.

**I: Okay.**

R: So this one is a bit different, we integrated the ART into OPD

**I: OPD okay.**

R: So, uhmm, I think it is a good thing to do, but I think we could start with sensitization, in the community, our patients, so that they really know that whoever has that condition and develops that condition whilst (unclear speech) can be seen, can be seen at the facility.

**I: Can be seen uhmm.**

R: Uhmm, I think it will be also a good thing for us working into [/in/] OPD you know sometimes it is cumbersome a patient comes from side no they have told me to come here. They feel we have to treat them like an emergency.

**I: Yes.**

R: So I think that will be a good thin from our part, it will eleviate our burden, so that at least we look at it 50/50 with the same thing.

**I: Okay, so you talk about previously when you integrated, how, was it successful, bringing the ART into the OPD and maybe if there were challenges, maybe if you can share with us a bit.**

R: It was not easy like at the beginning, people were not able like, not able to adapt to the adjustment to the new environment but later on it worked properly you know. Only know that we could have problems with long waiting, because there when you integrate ART into OPD it means you could have Malaria, diarrhea and all those things.

**I: Yah, all the things.**

But I think this one is a bit easier, simpler because we have the same people that we take care of and then we just add some few diseases that, so this one is much easier than integrating ART into the OPD, this one we integrating some few OPD into it.

**I: OPD into it, thank you very much for sharing those lessons with us, yes.**

R: Me

**I: Yes, yes (Sighs)**

R: No I feel, it is a good idea, it is a good thing, in that it will help with the time that the patient would spend at the hospital like you have always been saying at the beginning of the presentation. Patients will not be able to move from this point to the other point but will be able to get the services at one point which is a good thing.

**I: A good thing okay, any other? Yes ma’am (Chuckles) Or should I come back here, you are still, so I am coming back there and here yes.**

R: Yah actually it is a good move at least patients will not be moving from one department to another yah, so we will be explaining as they come, we give them the service and explain the reason why we are doing this same NDC [/NCD/]

**I: Alright thank you, yes.**

R: Personally I think people will, people on ART, this is good for them because they will be able to manage with the NCDs, so I feel them being managed with the combination will be easier for them and then, the only thing I think that should be done is sensitization to teach them to say this is what is going to be done, the new thing and also talking about the resources that will be brought in.

**I: Okay, alright thank you very much.**

R: I also feel it is a very good intiative of doing everything in one room, because it will strengthen total management of the person

**I: Okay.**

R: That’s what I wanted to say.

**I: Thank you very much, okay so I am starting from this side, yes ma’am.**

R: Okay, for us anyway it is a plus, because when we are sending clients to collect drugs from the other side, meaning they will have to maybe fail again to get drugs. So when they are already this side getting all the drugs it will be fine.

**I: It will be fine.**

R: Though the challenge maybe, where we have to give a bed, knowing that our area this side we don’t have space for beds. So maybe that way we may have challenges in some way.

**I: Okay, thank you very much, yes sister.**

R: Okay, me I would want to say maybe they should be an emphasis on IEC so that the patient understands, because there are those that don’t understand. Especially when it comes to patients who are diabetes and hypertension. So like the IEC should be there like each and every time we give these clients so that they actually adhere to the drugs.

**I: Alright thank you, yes sir.**

R: Okay, so mine also was in line with ICE, so I think as much as we introduce these services, I have noticed most of our clients have got their conditions already diagnosed. They know they are hypertensive, sometimes they try to refuse because they don’t want to be on another medication so they don’t seem to understand to say, it could be easy and it is manageable, just like they have managed to take ARVs. We really need to educate them, so that they understand and can actually do it (unclear speech).

**I: Yes sister?**

R: The intervention is actually good, and everything that I will do is explain the complications that come with NCDs so that they continue taking the medication none stop.

**I: Yah, alright thank you very much. So I am going to go back where we had left here, there then I will go to the front, yes.**

R: It is a good intervention cause mostly I work in viral load, so we had challenges, people with, clients with high BP, we used to take, send them to OPD so now our clients will be treated in the same premises everything will be there.

**I: Okay, thank you very much, yes I am back.**

R: Okay I think it is a nice thing, cause we used to send people from ART to that side and people they will be getting tired from that side. And then if you are sending them from ART to go up there, which means, they will not go up there, they will just go home because they will be tired.

**I: Okay, alright, thank you very much, I didn’t reach that, this side huh?**

R: Oh yes, I think ahmm, this is a value added services for ART and this will help our clients even in terms of retention. Because if the client knows that if I go to this facility, I am going to be seen for everything, it makes all of them to plan well. Then they know that I have to go, if ahm for diabetes I have to go to this facility and the other day I need to go for ART, you find that even retention is suffering because of the same. But if they can (unclear speech) in one facility it is even better but one would want to record is that we need consistency in terms of medical supply, because remember our clients whatever they get from ART usually it is free, they don’t have to pay anything. But if we have a service maybe that will be asking them to say we start doing the maybe for diabetes and we have to ask them to go and pay somewhere for such thing (unclear speech) it will be a burden in terms of the client. Because they will be used to say that when I go to ART everything that I do there is free of charge.

**I: Free of charge okay alright thank you very much? Are you representing the sister in charge?**

R: (Laughs) I am representing the sister in charger (Laughs) Okay so ammm, it is a very good initiative it is a very welcome thing especially that you are strengthening what is already existing and it is also a wake up call to most of the stuff, especially that most of the time we tend to ignore most of the Non-communicable diseases. So it is a good thing that it is coming on board to also help us sharpen our skills and everything to apart from ART management. Then also aummm it also help improving the quality of (Car hoots) service delivery ART usually clients when they have got a problem they will come through, so as they come through to complain about another problem they will also be helped in the ART aspect itself. It is going to be a lot of value and service delivery.

**I: Okay, thank you very much, thank you very much (unclear speech, whispers)**

**I: From our part we are done, thank you so much, we don’t know who is giving us our closing remark from the facility (laughs)**

**I: Synonyms (Laughs)**

**I: Sorry the matron excused herself (laughs) whose the second in-charge, sister Mwiinga please.**

R: (Laughs) We just want to thank you for your coming, as for me personally, the other part I was just thinking just now, this program has never happened from the time I have been doing this. I have never seen people come to do a study from this particular (unclear speech) actually I am very happy to see that people are coming to do this to do maybe even (unclear speech). We hope for a positive response from the community, otherwise we appreciate, we hope to work together as a facility and see how we can also help ourselves, thank you so much.

R: (Clapping)

**END OF INTERVIEW**
