# Supplementary material for: Applying human-centered design to adapt a multifaceted implementation strategy for integrating HIV and NCD services in Lusaka, Zambia: Healthcare worker perspectives
Source: PLOS Glob Public Health. 2026 Feb 2;6(2):e0005879. doi: 10.1371/journal.pgph.0005879 (PMC12863476; doi:10.1371/journal.pgph.0005879)
Supplement: S2 File — (DOCX) [file pgph.0005879.s002.docx]

**Interview Date: 24^th^ January 2022**

**No of participants:**

**Site: Chilenje Level one hospital**

**Interviewee category:**

**Interviewer: Tulani F.L Matenga**

**Transcriptionist: Tulani F.L Matenga**

**Time:**

**I: Please go ahead, yes Doc**

R: Yah, uhmm, maybe just the availability of testing kits lets say, lets say maybe diagnostic kits, lets say (Unclear speech) and all these in place, maybe that’s when this can work better.

**I: So you even have availability of commodities huh?**

R: I wanted to say the same thing, availability of commodities, which includes drugs, so we should have a flawless, supply chain, where we are not running out of this medication at any given point.

**I: Okay, thank you, again, before we get back to our discussion point, one of the things, that we have realized within Lusaka as well is that some facilities may be providing care in an integrated manner. Where clients are seeing holistically, the same provider manages your ART and also you NCDs, it is happening in some facilities. But then there are some facilties that are providing care separately, us we are only doing HIV, sugar you have to go to OPD and for some of these facilities, it means that me the recipient of care, I will come, sister Cecilia you manage me I get my drugs, then now I go to OPD a long queue and now I am at the back. So it means I am spending another 3, 4 hours, if I am working for the Indians in Kamwala it is problems. So what we want to find out as well is, in terms of challenges, what challenges or barriers, do we face as health care providers to provide care in an integrated manner? Or What is standing in our way, in being able to integrate these services? Yes.**

R: Yah, so basically, mostly you find that uhmm, at the moment, services are being done, you find that clients who come with hypertension they have some drugs in the clinic there already, in the pharmacy they have some drugs. But you find that you just have one particular drug, you cant supply like maybe those who are on, other hypertensive drugs, you don’t have. So that shortage, sometimes you find that, you might request, there are no drugs even in the main pharmacy. So maybe just the availability of drugs, that has been a challenge and diagnostic kits, space if we were to integrate then we will need space. Maybe we will need to monitor someone who has high BP, so we need to keep that person, so now where are we going to keep that person, while the screening is being used for screening other clients.

**I: Uhmm**

R: And uhmm, the other one is strengthening the Triad again. So we would like, like to have, we would like to have someone on the triad, who knows who is who, what do we need, this clients, what does this client need at this point. Who needs immediate care, who can wait and all tat, so its just to strengthen the Triad, space and availability of commodities.

**I: Okay, so those are the barriers that you are currently facing, in trying to provide care holistically right?**

R: Yah.

**I: Okay, any other input, (Pause) I want to hear uhmm, I need to hear your thoughts, or experiences, you can even share experiences, that can help at least strengthen the study itself.**

R: (Coughs)

**I: My brother there has talked about the fact that medication is a problem, so you cant really look at it holistically, cause there is only one drug that you have, so you end up telling the person to go to the other side and get the drugs. Also you don’t have the testing kits and also he talked about the Triad, so we have people there who know what to do, who know how to go about things, any other? (Pause), I can see you want to say something.**

R: (Coughs)

R: Uhmmm, okay like in MCH we are trying, yes, with the Doctors from the Eye (Unclear speech) so they are always there. And so if the mothers have any other complaints or even the babies, the Doctors come in to, to screen them but also it’s the issue of some testing kits like the glucometer and stuff, but otherwise we are trying.

**I: Okay**

R: Yes we are trying to do everything, and also its just that uhmm, they cant get the other medications because at the moment we only have like antihypertensive drugs and ARVs.

**I: So under MCH, because you are saying you are managing and you are trying to integrate services so what are some successes, or what are some of the facilitators, or we want to hear the success stories, from the, from MCH in terms of integrating services?**

R: Okay the challenges like uhmm, when we have uhmm, the diabetic, a client, we are just forced to take them to OPD, of course they will be accompanied by a Nurse and make sure that everything is done. We don’t let them go alone, so that they queue up or anything, we make sure that we escort them and everything is done.

**I: Okay.**

R: Yes and then on the successful part, those with uhmm, hypertension, the raised Bps, we manage them from MCH, we give them medicine, we keep them, we observe them, if they improve they go home. Those who fail to improve, we still take to OPD for further management since we close at 16:30.

**I: Okay so in some way you are also sending people to OPD?**

R: Yes (Laughs)

**I: Sometimes, thank you very much uhmm, any other? I will start with sister Cecilia.**

R: Yes, in addition to the management a clinician may request for some investigation like ECO uhmm when they send a patient just within the facility Chilenje you find that they are not doing such investigation, so they will end up going to a private institution to do those investigation. Then they will come back to say they don’t have money to pay because there at the private, they will request them to pay, because that one has been also a challenge.

**I: Okay so a follow up question for Webby, so have we lost recipients of care in that manner? Where we say if we don’t have, then the patient just says okay forget about it, have we lost some recipients of care because we are not able to provide such services.**

R: Yes. (Phone ringing)

**I: Any experiences? That maybe some would like to share in terms of losing recipients of care because we are unable to manage their NCDs? (Phone ringing) Any? You had young hands risen up.**

R: Yah, the other thing is with ART I think we are very good with record keeping, cause we use SMARTCARE. So you find that, for example I can see someone a patient in ART who was seen maybe by sister Cecilia even years ago, I can still, I am able to see. But with OPD, they are not consistent with using SMARTCARE, so you find that, for some NCDs, you need the history, the previous history, but if you cant find it in SMARTCARE it becomes a challenge for you to see them. So in other cases you are forced to send them back to OPD. But for those where you have been observing them, maybe every time they come they are hypertensive, let me say they are hypertensive, for those we do manage.

**I: Okay, alright, thank you very much, so before I go to the discussion point, I want to hear, because I know that we have main ART and DSD. So maybe just to hear some comments from our colleagues from DSD. Uhmm Malumbo is it? So maybe just to hear from you, I know, one of the concerns that we have from uhmm our recipients, recipients of care is having to spend more time at the facility. And I know that DSD one of your end is to ensure that recipients of care are spending as little time as possible at the facility and they can go and continue the rest of their day, so what is your, maybe your comments on some of the aspects that would like to introduce uhmm. Because of course, our recipients of care are going to be enrolled from both main ART and DSD.**

R: Uhmm two comments I would like to give, uhmm, to start with the management of NCDs (unclear speech) DSDs and uhmm whoa, I am giving this from point of practice that had been done before so, to start with there has been a shortage of commodities in the facility. Like he said, drugs is a very big issue, if you actually had to go in the DSD pharmacy we have incorporated a pharmacy in there so that it is like a one stop shop. But we are limited to the type of drugs that we stock, its not every drug that we stock and most of the drugs are not even there at main pharmacy so even if you send somebody to go and collect drugs from there they will still send you back and say we don’t have. Secondly it has to do with space, this is why when somebody starts reaching a certain level of illness we fail to keep them because we know they need proper care in other departments, space, like he said, somebody comes and who it’s a critical patient, the BP is high we don’t have space where we can actually keep them and monitor them. There has to do with space as well, so there is drugs and space.

**I: Uhmm**

R: Secondly [/thirdly/] you mentioned incorporating this in SMARTCARE where it pops up and stuff like that, uhmm whoa, it is a good thing and it can work out. I just don’t know how best we can do it, because one of our indicators in DSD is we don’t keep certain comobilities, we cant keep them at DSD meaning we need to drop them. So I see a certain where main ART might be the major lead for this, but even them that means there will be a lot of work to do there will be work overload for them. And one thing I have noticed with SMARTCARE every time you introduce new patch in SMARTCARE you introduce this, it has a lot of glitches. Just recently they introduced a phamacovigilance batch and it started deleting some other interactions. So I don’t know how one am sure one, I am sure sister Cecilia will speak on this, I don’t know how we could probably help with drugs and probably also stuffing at at main ART. Because I see a situation where they are going to have a lot of clients at the same time. And thirdly is I don’t know how best we can also introduce if it is possible, introduce a pharmacy in main ART as well where they start stocking uhmm essential medicines because at the moment to my understanding they are only stocking ARVS is it?

R: Yes

R: Yes so I don’t know if that can also be advocated for where they also start stocking essential medicines.

**I: Okay, thank you very much for that, so one of the things we learnt is umm, our colleagues from uhm, uhm medical store, cause remember I said we have a lot of moving pieces. So we have a group that is looking at Lab, we have a group that is looking at pharmacy the idea of course is each group strengthens their area within the facility. So one of the things that we heard is the drugs are there, medical stores, so what are some of the challenges here apart from availability of drugs? Is it the procurement system, what exactly are some of the challenges because as medical stores, they are saying we do have the drugs and some facilities, and some facilities do have some drugs in bulk so I want to understand the procurement system. Maybe, maybe some of us will not be able to know what goes around, but do we have any issues that need to be strengthened in terms of procuring the drugs. Because from our understanding with interactions with some of our colleagues is that, these drugs are there, right, at medical stores. Sister Tenda, from some interactions right, we know that drugs are there at medical stores and in some of other facilities they do have the drugs that we are (unclear speech) as well.**

R: Yah so for the drug component, medical stores tend to have drugs in stock and we don’t receive requests from the facilities. How do we just push drugs when they have not requested from them.

R: Medical stores.

R: (Cross Group Talk)

R: Medical stores, you push it to me so that I can put it in the terms where we are just, we understand.

**I: We understand yah (Cross talk)**

R: Medical stores claims that they have some of these drugs, in stock, but they don’t receive requisition from the facility why should they offload more drugs when the others are still there in medical stores. So what he is trying to uhmm to get at is, we want to see, where is the challenge? Is it us on the requisition part that we don’t request for these drugs and if it’s uhmm we hear from the pharmacy part, and if the pharmacy part will give us, their view, we will be able to note, so that we revert as well, and from there we will see, where is the gap? Why is it that some drugs are there but they are not being requested, why is it that the pharmacy person, not, our pharmacist why is she not requesting for these drugs, then she will give us also her feed. I think from there we can pick it up,

R: She will pick it up.

**I: So we have, the boss of pharmacy.**

R: I can feel her eyes that she is just (Laughs)

**I: So we want to hear from the boss, medical stores says we have the drugs, we are here and there no drugs. So we want to understand, how do we come in to strengthen some of those systems, do we buy drugs again? Or we strengthen it, just somewhere, somewhere, so just, can pick it up.**

R: So we do our orders on a monthly basis, through our R and Rs, ehh, that is ELMS, “Electronic logistics management system”. So when we do those orders, MSL when they deliver, they only deliver a few products. Even when you follow up by phone call, you ask them about certain commodities, they wont have. The bulleting I received last week, hypertensive drugs, months of stock was 0.1% which means if I do an emergency order today, I might not even receive those commodities. It maybe they have already given them out to a certain facility and Chilenje wont be part of it. So I don’t know maybe we need to invite MSL we come and have a chat with them so that instead of us speaking, pharmacy is saying this, MS, ZAMSA is saying something else. We need to sit down and synchronize everything.

**I: Synchronize everything okay, what, in what, maybe you could comment on some of the areas that we could strengthen. Do you feel that we need to come and strengthen the system that we need to procure the drugs or do we need to be there to provide, of course we are going to provide a few drugs here and there, but we do understand, it is not sustainable for us as a study to provide. Because remember what we are saying is we want to provide evidence and give it to the ministry of health and that is what should be happening uhm, over a long period of time. But we also understand like what should we do, how should we come in temporarily to ensure that we sort out this particular challenge?**

R: Okay, so for a start when we do our orders with ZAMSA, if they don’t have those commodities, I think you may need to come in and do local procurement on our behalf. Just to buffer up until such a time that if they promise in the next 3 months they will have, then they will come in and do that.

**I: Okay.**

R: So you need to come in to buffer up the stocks that will currently be available.

**I: Okay, alright, great, so I think we will be (unclear speech) we have a team pharmacy, people working with the people that are in that. In sort of trying to strengthen the facility’s procurement system, trying to understand as well, what are some of the drugs that are not available.**

**I: Just a follow up on that, when we do our requisition as pharmacy do we uhmm, request just as pharmacy or do we request as uhmm, as from demand? When we are doing our requisition as pharmacists from is it medical store is it?**

R: ZAMSA

**I: Yah, ZAMSA, do we request as per demand, or do we request as pharmacy (Unclear speech) haven’t spoken, is there someone who wants to?**

R: So when we do our order, we do order for everything, so the electronic logistic management system is in such a way that even for things that are slow moving, it will still, you still order for those. Even for things that are currently not in stock you will be able to do your order on them.

**I: Okay.**

R: Yes so we order for everything.

**I: Okay, because sometimes I look at a situation whereby because I am not receiving any prescription for this commodity, why should I even put it? Because they are going to expire on shelf, that will be another case on me. So I was looking at that angle, so do I demand you know? Yah, that is what I was looking at, because I ordered a few of these but since 3 months, they are still on shelf, do I need to order them?**

R: You still order them, so you order what is enough for your facility and in a situation where you are unable to finish them. Then you communicate with either DHL or whichever PH, they will be able to share with other facilities that have more clients than you what you have.

**I: And then at the facility level, like at our level, do we send a bulleting to the department to let them know the commodities that we have. Like in terms of drugs that we have? These drugs, cause sometimes you might have in stock in pharmacy you are well equipped yah?**

R: Uhum

**I: But I go to ART or I go to OPD, they just ah, we ordered 3 months ago, there is nothing at the pharmacy, they told us that there is nothing. But meanwhile, you have done your part, it is there in stock, yes you continue ordering, even that month they told you, the other month they told you, the past 3 months they were telling you it is not there, but finally it came and it is there. Do we update the department to say, these are for this month, we have these commodities?**

R: Only when we do management meetings so the heads of departments will be informed. So we need to create, I think we are working on that, creating another platform from where we will have the Clinicians, the Doctors, the Nurses that are seeing the patients should be included in that group, so those ones will be updated weekly.

**I: Sister Cecilia do you think that will be helpful?**

R: Yes, very much

**I: Dr Mwiinga?**

R: Definitely.

**I: So like we have come here, we are saying, I can’t think of what Chilenje really needs and just bring them that, but I need to hear from them, then I will be able to see where do we meet so that we are all comfortable, yah so that is where we will go.**

R: Yah, so just on that one I think, one practice I saw at Chipata general Hospital where there was, we use weekly bulleting from all the drugs in stock. So that can help, I personally haven’t seen one here at Chilenje I don’t know if it is done or not. So all the clinicians in whichever departments will have access to the weekly bulleting of all the drugs in stock.

**I: Okay is that your, sort of suggestion to us, to say the people that you work with, they should be updated with what is available on a regular basis?**

R: Yes, so that even as I prescribe I know amoxyl is not available but we have stethroxyn as an alternative.

**I: Alright, okay.**

R: That is what I wanted to say.

**I: Thank you very much**

**I: Can we also suggest to the HOD to say, would we have these drugs available as well? Is that possible?**

R: Yes that is what we normally do.

I: Okay, alright, thank you very much, so one of the last, one of the, before I discuss some of those, one of the things I want to hear, I think over the years, the issues of sustainability are a very big part of, of, donor funded projects, in low and middle income countries. What we are trying to avoid as a study is, that is why our team is a very small team, it not made up by a lot of people, each one is heading is a small section. Because we don’t want to see a situation where we come, we provide evidence, then we say we leave you have to take over and then you say that study that used to do that left. So we are going back to what we used to do, what we are trying to say is, how do we sustain what we are proposing? Because it is going to be given to Ministry of health, it is going to be given to your facilities. When we do the pilot, for enough, for about 5 months, we want to roll to most facilities and come back and give evidence and say this is what works, but then my question is, how do we sustain HIV/NCD integration beyond what we are providing, beyond the support we give you in terms of purchasing some drugs here and there making some testing kits available and also having a few stuff here to support you, training you as well in managing both uhmm, conditions and also ensuring that the SMARTCARE system is able to incorporate efficient and managing to have data on both conditions and sustainability. Sister Cecilia I will pick on you because you are the boss, (chuckles)

R: On that one I think on the program among us as medical practitioner, I think we can pick one as a champion to spearhead the program. So that champion will be responsible to move round from ART, in-patient also OPD and casualty, at least every week to do most of the reports.

**I: Sister Penda you’ve heard we need to have a champion who is spearheading this HIV/NCD integration to ensure that this person is pushing the agenda as well, my other suggestions?**

R: And also if it can be added on the dashboard, the one which we have weekly, also for the month data.

**I: So (unclear speech) we also need to become, like to become part of your monthly reporting, so it becomes like it’s just the norm, it is part of the system, alright, any other? We want you to suggest as in many, because remember you are changing so, you know how these projects are done, we sit somewhere and read a lot of things and put things together according to what we feel is right. Because we don’t practice, we don’t really know how things should operate. So we want to know as much as possible so one of the last questions again is, sorry to take you back. If I said uhmm, because remember this is our suggestion, we sat somewhere and developed all concepts, but if it came to you and said, oh my brother, we want to design and intervention that is going to ensure that both conditions are managed or looking at recipients of care holistically, how would you design it? Those 5 components are what we are, talking, we are being, myself, sister Penda, our PIs there at HQ, this is us sitting and just thinking. But for you who is on the ground, how would you design this particular, or with what we are suggesting, what would you take out, what would you change? I come to say okay now design, so what you are going to say, is what I am going to do now. Malumbo? Any of those suggestions, one of those 5, what would you remove, if I told you this is what you design for this intervention, what would you add? Does it even make sense? The 5 components we are suggesting, integrating one of, umm, making available testing kits, all those different components, task shifting, or in short how would you design an intervention that is looking at recipients of care that have got both conditions? (Vehicle noise)**

R: (Long pause) My suggestion would be not really taking out anything, it would be, at the end of the day we are looking at patient welfare and at the end of the day regardless of what we are trying to incorporate we are looking at how good the patient is going to receive the service and for any integration that comes, maybe I will just love if you introduced a patient feedback. Where uhmm, we get feedback from the patient, where you know, even as much as we try to care for these patients and we are trying to manage their NCDs, most of them are ART clients, some are busy, some might not be interested and this is not really a forcing matter and the only way we are also going to improve is to get feedback from patients. So I don’t know how best we can introduce that component of feedback.

**I: Alright, so you would add on a component toward getting feedback, as we going about this integration, we need to know if it is happening right?**

R: That is after the patient is done with everything at the last point, then they give us feedback, or where they think we didn’t do well or stuff like that. Then secondly, is I would also want maybe to introduce uhmm, need to introduce trainings as we go on, that’s frequent training, after a while, You know people have training, especially our community health care workers, because these are key, and mostly have a role [/lot/] to play and interact with the clients on a daily basis so they can be having form of these small trainings as well, that could also be helpful.

**I: Alright, thank you very much for those insights, so we have Doctor Telo on the team, Dr Telo is the one whose designing the SOPs and the training material, so Doctor Telo, we will be meeting her I think as we proceed. She will come in to ensure that we are trained in managing both conditions, so you will be meeting her as well. Cause remember we said that, we are not going to provide care for people ourselves, because we are not medical Doctors, we are just there to support you, meaning if you are saying you need a training, this training will be made available. You need the medication we will support you in that way, you need SOPs, we will support you in that way, so basically, it’s you as providers, who are doing what we are proposing in short. Yes?**

R: I have a question, as we are managing these clients in ART, let’s say today is their appointment they come and we find their BP is high and we manage it, so the coming day, let us say, today they come they go home, a week later, the appointment for ART is in 3 months, one week later, their BP shoots, are they coming to us, or they are going to OPD?

**I: Okay alright, so the idea is now, we have taken them on at ART, so they are not now part of ART, because remember ART is part of, we are going to ensure we have everything, remember its one shop right? One shop, one stop, one record, so I cannot go, I should not go to OPD it means I should return to ART because in the ART clinic, there is you who can now manage because we have gained the capacity to manage both conditions. You are doing everything at ART and because ART created a relationship with you, you have been on ART for the past 10 years, so I know, you know my drugs, you know my problems you know all those things and you might even know where I stay where you can call and say eyyy, how are you feeling right?** Yes.

R: Are you also going to incorporate community dispersion sessions (unclear speech) because we have our friends who collect medication the community and they might also have these NCDs, so are you going to incorporate the community dispersion does that mean you would have a professional follow when going to do this dispersion to see this patient from home and disperse, I would like to understand how that is going to work.

**I: So for the community unfortunately we don’t have a very strong component of the community aspect in this particular study. But these are some of the things that we would like to hear from you. What would you suggest do you think that it is a good idea? If it is a good idea, who does it, how do they do it? Those are some of the things that we need to get feedback, we don’t have a very strong component in terms of community dispersion. But we do know it’s been successful in the ART clinic, so what we are trying to do is to liverage the success that we have in the ART clinic, can also bring them on the other side, so these are some of the things that you say we should bring on board, then we go back and say redesign and reconfigure things. (Paper flipping)**

R: So if uhmm, this program, is not just looking at medical emergency at that stage isn’t it? Let me, I stand to be corrected, so we are looking at this person, who is HIV positive and has a diabetes or hypertension or both diabetes and hypertension, whether controlled or uncontrolled, isn’t it? So if we say we have to do this holistically there is a certain stage where our clients, for example are unable to come to the clinic, to the hospital, for one reason or another. So if they need to collect, those medicine are also taken for a life long period, so if they need to collect their medication, there is no way we are going to deliver them ART, and then we don’t deliver them hypertensive or diabetes medicine, then we ask them, no for the diabetes medicine you are coming to the clinic. So if we have to incorporate it means that even as we deliver our ART medicine, we are also delivering the hypertensive or diabetic medicine.

**I: Alright great, thank you very much, so I think that is a proposal I wanted to hear going forward.**

R: On the previous point where you are saying for some screen visits they will still come to ART, I feel that will cause traffic in ART and the whole purpose of trying to decongest and make them not take a long period of time at the facility will be beaten.

**I: Okay then, what would be your suggestion if someone is coming back, uhmm not to collect ART right, but then for NCD, what would be your?**

R: I think the usual of just going to OPD is fine, I don’t know what other people think.

**I: Going back to OPD? Any thought?**

R: I am thinking cause if we say, we will incorporate the two and then we check there is still continuous care whenever they come even if it’s not their appointment, that will put pressure. One, you find that maybe the screening rooms are not enough to see the expected list and to see those who have just come to manage the blood pressure and then manpower also, I think that might, be, might have pressure. So it will need maybe a separate person to be screening those maybe who are just returning for Non-communicable diseases and others are seen those for that particular day.

R: I want to understand one thing, I think I am also seconding Dr. Mwenya and Jacob, so this is working best when a client is only coming to collect medication, and they are only coming, coming to collect say, they are hypertensive and dehypertensive ART client, so they come to collect the 3 months or their 6 month and collect their hypertensive. But in times when they need proper care, because remember these clients, will also need care sometimes, they will need to be seen, they will need to talk so somebody, their vitals need to be checked, that is also adding up as another service. And uhmm, in times when they also have an attack in the middle, probably they go and in a month or 2, they need to come, especially if it is a client that needs to be monitoring their BP, it means they have to be coming back, even when it is not their appointment date and that might bring a lot of traffic and congestion at the main ART. So the alternative would be to send them back to OPD, but that doesn’t still come back to the same thing of them just going to OPD.

**I: Okay, I get what you are saying, I think that is one of the things that we will look at when we start to pilot test, is how is traffic. I think another component we are going to look at is time, how much time a recipient of care comes, how much time do they spend from the time they enter the facility to the time they leave the facility as well. So we want to ensure that we also track that so that we avoid our intervention are not being like an oral but on the recipient of care.**

R: I will give you that, at DSD it is one hour, that is the maximum is going to spend in the facility, everything is done, (unclear speech), the clinicians, they go to the lab, come to pharmacy in an hour they are supposed to be out of the facility, that is one of our (unclear speech). So if we are going to add up another service, where, you know, it might touch on our indicators, I don’t know how best we are going to (unclear speech)

**I: Okay, so I will keep your question Malumbo, first of all I just want to ask, I think we talked about overburdening health care workers, so the first question, I want to talk about, the discussion point, how might we integrate a TASKPEN package into ART clinic work flows to not overburden the health care workers and make patients wait for too long. So how do we do this, without one, overburdening you as health care workers who already have 99 jobs and how do we ensure that we don’t keep recipients of care for a long time? So you have come, the question was asked to me, now I am asking it back to you, so what we want, your suggestion, how do we ensure that this we don’t overburden ourselves, two, we don’t spend a lot of time with the recipients of care. So this is you now suggesting, some strategies we can use.**

R: Okay, uhmm, looking at if, lets say a client comes, the BP, is high, we manage, we control the blood pressure, and then maybe the next time the client the BP is high, in between before the appointment, maybe they can be seen in OPD. We can attend to them on a particular date on their appointment, when they are all those happening, like they need more supplies on their medication and everything, it can be done at once. But when they return they want to come back in between, they can maybe access the service from the OPD.

**I: Okay.**

R: The other one is maybe we can just stabilize and send to OPD

**I: and send to OPD (cross talk)**

R: More like it is an emergency, you can just attend to what is happening there and then we refer.

**I: People have got different view, (chuckles) and then on overburdening health care workers, how do we ensure that we don’t overburden staff already that are doing 99 jobs?**

R: Put more staff.

**I: Put more stuff (laughs) I like that, put more staff, (chuckles) so we will move like this.**

R: So uhmm, I think the whole purpose of incorporating is avoiding for example avoiding a client from, I see the client as ART, isn’t it, then they go to OPD to collect their hypertensive drugs. But there are times whereby, one thing we should remember is these clients don’t come around Chilenje, there are clients that come as far as maybe Garden house and what, what. So if we are providing them with this service of incorporation and in between their BP is high, we usually advise the client to go to the nearest hospital. Remember the client is spending money, transport to come here, so they cant spend transport, just to come all the way from Garden just for BP check up in Chilenje, when there is a clinic in Garden house where they can go and be managed for that hypertension, I don’t know.

**I: Yah, so you are bringing in a very interesting dynamic, isn’t it? There are those that you have told to go nearby, if someone is coming from far, should I always be coming back to Chilenje because that is where I am receiving integrated care? Sister Cecilia, there was, oh you had.**

R: Uhmm, to my part the pharmacy we are really under staffed so if we bring in that initiative you need to help us with manpower when it comes to the pharmacy.

**I: So issues of manpower?**

R: Yes.

**I: It is good that the boss is here (Group laugh) (unclear speech)**

R: Yes I agree with him, it is not just the pharmacy, also on the Nurses, the clinical part also, there is little to no manpower. And in addition to that, I wanted to find out if you can do some extension to the PREFUB, so that we create some more rooms, so that patients they are seen at one point, not whereby, if they come for, uhmm, drug pick up specifically for NCDs, they are not being sent back, to casualty. How I wish it can be done just there, but the space is limited as at now.

**I: So we need space, okay.**

R: Some extension, can work

**I: So I will go to him, then come back to you Malumbo,**

R: Coming from casualty, since I have heard a lot from the ART (Laughs) let me look at it from my side also as casualty, I am just concerned cause when we look at these NCDs, like, I am trying to hear from ART, the extent of services that we are going to give. Because I am just hearing about medication and hypertensive, but then we will look at conditions like diabetes, there will be times where someone might not just come with hypertensive, they will come in DKA. So that will take a lot of time, because it will be more than one hour or five hours, so that’s what I, I am interested in knowing, if we are going to say, we are combing and integrating, like how, to what extent are we going to be offering these services of NCDs and ART?

**I: Okay, so let me get a question from there, then we can discuss that.**

R: Uhmm, it is just a suggestion, I see this working, but I see a situation where can’t run away from OPD. I see a situation where we have to have OPD on speed dial, because to my understanding, we are trying to still fast truck these clients isn’t it?

**I: Uhmum [/yes/].**

R: These are ART clients we are trying to fast truck them and I see a situation where we have a lot of limited space to start with and you know it wont just come overnight where these extensions start happening. But is there a way in which we could pick individuals from OPD that can be on speed dial as uhm, specialities. Say for example we have a clinicians at OPD or a Doctor at OPD in times where we know that this client might need a lot of care and we are a little bit busy, we can have somebody fast truck them at OPD, seen at OPD and dispatch from there and they can leave the facility.

**I: So we are talking about having people at OPD, who will be ready to attend to clients, right?**

R: It is like they are part of this, so it is like already a specific person needs care, a CSW can quickly escort this person they are seen and.

**I: Okay, so we need sort of someone, uhmm, sort of another champion at OPD who is part of HIV that’s your suggestion?**

R: Yes that’s my suggestion, I don’t know how people take it.

R: Alright, follow, them up, mentioned of space, they might not come like (Unclear speech) yah so hopefully casualty might have space. So if that person is sent directly, to that space created there, the patient might not take long, like the way, they start afresh (unclear speech) that side

**I: In addition to that there is an interesting question that I want to ask, I know, I know recipients of care said, they will bring the ART Clinic, I mean bring NCD management into the ART clinic right? What if we said okay we are going to have a separate space where we are going to have recipients of care with both conditions coming, but we know we have ART clinic, so you know, I am on ART, so I am not going to go anywhere else, I am going straight to the ART clinic? Uhmm, what do you think about the different space somewhere in the corner, like oh managing recipients of care that have both (unclear speech). As you think about that as well, I have got only 2 last questions, the other one is, how can we encourage treatment supporters and community health care workers to take on the extra task of educating people living with HIV and NCDs and tracing patients who miss medication. So how can we encourage treatment supporters to take on this extra task that we are suggesting? Following up in the community, educating ahm, ahm, ahm, people with both conditions in terms of DSD drugs and lifestyle changes?**

R: (Unclear speech)

**I: How can we encourage them, what do we do?**

R: (Group Laugh)

**I: I want to hear from you!**

R: Motivation

R: Motivation

R: (Group Laugh)

R: Money

**I: How can we encourage?**

R: Okay, I think first thing, these people need to be trained.

**I: Okay.**

R: Yes, yah that is he first priority, they need to be trained, when they are acquire knowledge now that is when now motivation comes in. cause they are good at community mobilization these people, very good actually. All they need is, training and motivation.

R: Kwacha

**I: Any other, any other ones [/ways/] how we can motivate them?**

R: Kwacha.

**I: Just the Kwacha?**

R: (Group Laugh)

**I: Okay my last question.**

R: (Unclear speech)

**I: So my last question is, how might we encourage workplace environment that embraces NCD integration such as task intervention is accessible, desirable one and usable for facility staff. So how do we encourage, how do we make sure that for example Chilenje, level one hospital will embrace HIV/NCDs strategies, how do we ensure that ahm, there is accessibility for this particular service and staff are using it. So what we are trying to avoid is, we are supposed to be integrated right? So we have seen that in some facilities, like oh that facility is integrated, so when you go there it is the same, here it is ART only, its just medicine, those other things you are going that side, so now I am now moving from there, I am moving to the other side. So how do we encourage this environment where people as providers, we are accepting using that approach and it is very much accessible and recipients of care with both conditions if I come, it is holistic service and not back and both within the health facility, so how do we create this environment? This TASKPEN environment, Sister Cecilia, its good to be the boss, because will be talking all the time. (Laughs)**

R: (Laughs) I think it can be started from the Triad where the treatment supporters (Unclear speech) from the triad, the treatment supporters start giving the health talk before they start attending to the patient, and also when they are doing the vitals there, they will be able to see and differentiate when ah, they are being trained, that this is a raised BP. Also I don’t know if they are going to be doing the other RBS test, to differentiate the normal from the abnormal, so that they separate or they fast truck those who’ve got arnomalies to the clinician.

**I: So we need to start from just the entry point there, like information is given like we are, looking at patients holistically, then it flows all the way to the others, right?**

R: Yes

R: I don’t know I am just thinking out loud, the community health workers I think they do a very good job in the community, if I am in the community, this person comes, I stay with him in my community he tells me that Chilenje level one hospital is giving you these services. When you go there for ART you will be seen with your NDCs [/NCDs/] I think even as I am coming here, the first thing I want to see is where should I go? And I expect to receive everything from that facility.

**I: So information, should be there in the community, so that as people are coming they know that if you don’t see me holistically, then I should report someone that it didn’t happen.**

R: Yes

R: It is just a concern, as we are looking at it, have we put in consideration, the issue of confidentiality, cause I am thinking, I am overseeing something whereby, if people are coming to casualty or OPD with people that don’t have HIV. I am seeing a situation a situation whereby OPD or casualty will start seeing people after Traiding and then maybe they do the test, they will start telling people to say, you know what? No HIV or ART services for everything for hypertensive you have to go to ART. Then there will be that division whereby others will be saying why am I seen from casualty and these others have to be sent to ART but we have come here for the same problem. (Phone vibrating)

**I: Sister Penda do you want to come in there?**

**I: Okay, so I will revert back the one who has generated the question, what do you think can be done to avoid that? You are the in-charge for OPD so you know best how to handle each department.**

R: Because currently we are seeing, regardless, the way we are doing right now we are seeing both ART and non ART patients. So now if, cause now if, cause me that’s why I am concerned we introduce, we integrate now NCDs for ART, it is going to be mean they have to be going elsewhere for those services so I just, I am just concerned. Actually I am just concerned to say why cant we, continue doing it the same way.

**I: So I queue, at 06 AM in ART, I am a stable client Malumbo has given me 6 months, he is going to see me after 6 months right? My drugs, then you, you are only giving me, weekly or no drugs at all, meaning still, that effort which Malumbo has put in, to only see me after 6 months, but you, you tell me, you should be coming for BP everyday, you have seen right?**

R: Uhumm [/yes/]

R: Yes

**I: Which one would be better? Would be beneficial for me as a client? Because Malumbo there I am satisfied, my viral load is done, now I just have to continue coming for my BP and sugar levels. So basically this intervention, the TASKPEN intervention wants to leverage on the existing ART, I mean services, what do we mean? We mean if we are able, if ART has reached a point of seeing me once in a year so to say, once in a year, oh twice, twice in a year, after 6 months, twice in a year. Why cant OPD. But if I come in January I will only feel like the next time I don’t do that, why cant we also, reach a stage where, we stabilize these people with NCDs and they also start getting 6 MMD so to say, that is what we are, that’s our goal, that’s what we are looking at. Hence, because the government has no capacity to just pull in and we are yet to source for more funders, more donors, so that we can as well push the NCD to say this is also critical, so this study is trying to push as well because even when we started it was 2 weeks, 1 week at the most, 3 months ey?**

R: Yes

**I: But because people have now understood, they are educated, even when we just move, they like uhmm, you refusing medicine, they will even laugh at the others who are not taking that you will just die. So that is what we are trying to do, but for us we just cant come and say okay, there are all these gaps with NCD care, there are all these gaps, the drugs are not there, even just a glucometer, how much are we charging just for a glucometer?**

R: k20

K: K20 Kwacha

**I: K20 kwacha, (unclear speech) k20 just for that and then it starts, you find my glucose, it is 26, then you put these drips on me, but you need to come and check again to see how much am I going to spend cause you take 30 minutes after every 3o minutes (Unclear speech) how much am I going to spend? So basically what we are saying is, we are not saying that you should be chasing them now, you are HIV positive go that side no.**

R: But you see, that might happen at some point.

**I: No listen, it is you and me our mentality but that is why we have told you as a head of casualty so that you are going to see how you are going to handle these children under you, so that they don’t say, on ART, high BP, take them that side. We are going to now find a system, right, system on how we are going to handle this situation. We don’t’ want to discriminate, we have worked so hard on discrimination, such that we are saying it is there but not, the most which is there is self. Malumbo is around he will look down on me that even I have, self, not that, people will say, he is taking ART, so what we are saying is, that is why we came to discuss with you people on ground. We are saying we want the best way possible, how this integration is going to work. Of course, if I come, I am on ART, you are going to see me in OPD, the way you see me in OPD, because you also should know and learn about this TASKPEN intervention the guideline you would know, you are going to handle me, the way you handle pregnant women, they come to casualty isn’t it?**

R: Uhumm

**I: When you take, take, take, uhm, uhmm, this one is above 28 weeks, oh this one is over 30 weeks, this is a maternity, do you say, upon seeing me pregnant you quickly say, go to labor ward? No (Group laugh)**

**I: Huh, you would say, you look at me, you would say, you even call, Everlyn, Everlyn, you even call Everlyn, Everlyn kindly take this so mama, because of the issue with the pregnancy we are going to take you to the Doctor that attended to you but (Whispers unclear speech). I am going to feel discriminated? I will be very happy because I know that you are going to take care of me very well isn’t it, it is because you want to take care of me very very well, you have done the basics, you have even checked my blood pressure so that even as she takes me there, all those parameters are already there, the blood pressure, the Doctor now will know to look at my vitals and will concentrate on my pregnancy, so we want that, how best can we because me I wont come to tell you to say, Macdonald, let us work it out like this. You know the way our department works best, I can bring ideas, like I am bringing, I have given an example but you know how best it can work, you know how best my attitude as your subordinate under casualty is, so you would know how best it can, so that is why I reverted back to you to say, what do you think can be done, so that, so that we don’t encounter that. Because you have foreseen that, so we now want to ensure that doesn’t happen. Can we think about that and see how best we can avoid that situation, yes it can happen, but it will be a 1% of 0.5% we expect that we are human but we want to see how best we can.**

R: So mainly it should be profile orientation on the stuff I think before the program can be introduced.

**I: Okay, so I think in the interest of time, what I will do is, for you who have got comments, you are going to make 2 comments, so I will just go around the room, I know I have kept you over lunch, my boss here is telling me time (Chuckles). So I want, you will sort of comment, you can make 2 comments overall what you think about what we are proposing, but the most important one I want to hear is, we are going to be a team, that some of us have got no clinical background, thus trying to support in different ways. So what I am trying to ask is, how do we work together? How are we going to work together to ensure that we provide care, in an integrated way, what we are trying to avoid is, here do you see that guy from CIDRZ, he is just sitting and looking at me there as if he is my boss, we want, we want also to be integrated in the system we are there to support you, even though we don’t have any clinic background understanding your challenges and how do we move together. So I want you to comment on how we can work together in a coordinated approach and those guys, if you have, you can raise you hands and give any 2 comments or questions. So those who had their hands raised, so we are saying your comments and also how we can work together, then others can just talk about how do we work together. Then uhmm, I will pick the boss to speak last, then we will give some concluding remarks and then we can see how we move from here.**

R: Okay, how we can work together, I think I will start with you the people who are bringing this project, you can help us actually with space, commodities and also, trainings. Then among ourselves, it is about linkage, proper linkage communication when we are attending to our clients.

**I: Okay.**

R: I think that is all.

**I: Alright thank you very much, we will go like that (Pause).**

R: Uhmm, what am I supposed to

**I: I know you had your hands raised, so you can give the question on how we work together ahmm, cause like I mentioned, we are not, personally I am not a clinician, so we have got no idea of but then trying to be on the ground to support people that have the knowledge, how do we work together? And also you can give your comment, so we are taking 2, a comment or a question and then talking about how we can work together.**

R: I will start with, my comment, I have sister Cecilia and Doctor Mwenya in here and basically, I think when it comes to ART they might be in the best position to give a comment on how this is going to work because we report to them and they know the weaknesses we have in all departments, so as in lead, I would life them t actually give the official comment for me.

**I: Okay.**

R: Then my question will just be on commodity, I know it is directed to the pharmacist.

R: Madam Machizo,

R: Madam Machizo, I know this might mean, we might have to increase our consumption of drugs, not ARVs, because if we are looking at us dispensing 6 months of anti-hypertensive you know diabetes medicine, it might increase our consumption. Are we able to justify that to start receiving more consumption based on that? And uhmm, are we also able to have enough stock, so that we don’t reach a point where we have run out? We are able to?

R: I think before I came here, I had a brief meeting with Brian, you know him?

**I: The pharmacy?**

R: Yes, we had a long discussion on putting up something to ensure that the medication will be available for our clients at least for 6 months. And then they will have to come in where we are unable to (unclear speeh). So it will be continuous, I think ZAMSA has already been engaged (unclear speech) I don’t know how far, I don’t know, I need to see how far things have gone.

R: Okay, thank you.

**I: Thank you very much for taking the floor, on my behalf, so we have, so we have like we said, we are here to support you in our various capacities, as pharmacists, as people in the lab, some of us do qualitative. Trying to understand really the barriers and how we can support you, so we will be here for sometime, doing very different things, ahm, at the end of the day. Sister**

R: Uhm

**I: So we want to hear your comments of how we can work together, like I mentioned some of us are none clinicians, some pharmacists, lab doing different things, but then how do we ensure that we don’t interrupt any of the services, cause at the end of the day, we don’t want to interrupt, any of the services that you are providing at the facility.**

R: Okay, a lot of things have been said, but uhmm, I was just thinking anyway, like for the NCDs clients, uhm, honestly you cant get medicine for 6 months. There are times maybe you can get for 2 months, you can, you can get a standing, like a standing order prescription where you get medicine every 2 months, or every month like when you come with a prescription, you just go for medication you are not going for any interactions unless you have a problem. Unlike 6 months is given to this person and you know our rationing in the hospitals, this one gets 6 months then the others might get less.

**I: Get less.**

R: Uhmm, I don’t know, I stand to be corrected anyway.

**I: Okay, so we have taken all your views and ensuring that we sort of incorporate them, yes.**

R: Ahhh, okay, looking at the practice we have been doing, whenever a client comes, we always look into the history, but we have seen some files, where I cant see where the client was seen in OPD maybe, they have there the book and everything was prescribed, everything was there. The vitals, they were entered in SMARTCARE, and then you just find maybe vitals and you find there is no any other history on the patient, so maybe, that would need integration on people screening on the other end. If they can use more of SMARTCARE on what has been tabulated of what has been discussed on that client, maybe that would be helping on our end when clients come.

**I: Okay**

R: But I know casualty gets to be busy

**I: Gets to be busy yah (Cross talk)**

R: That we understand, but how are we going to do that, if maybe there will be someone to update after they see those clients.

**I: Okay.**

R: Yah, that can really help.

**I: So we are talking about effective use of SMARTCARE?**

R: Yes effective use.

**I: If we could even train people to uhmm be comfortable using SMARTCARE for both conditions.**

R: I don’t have any comments right now

**I: No comments?**

R: No

**I: Alright, thanks.**

**I: No, no, no, you will not say so (Unclear speech)**

**I: My boss has said no (Laughs)**

R: I think everything has been said.

**I: Or you can say something that has already been said (long pause) we will come back as you are forming your comment.**

R: Okay, uhmm, it is going to work out, as you have said, that you will be providing, drugs and some of the things to use, testing kits and every now and then. And then with us working hard, plus we need orientations every now and then it is going to work out.

**I: Thank you very much, so there is support for us here sister Penda, it is going to work out.**

R: So uhh, I wanted to make a point while we, this program I don’t think it is made to make casualty or OPD redundant, it’s, it’s a program where you are, for example the way we do things in ART. Where a client come, the client at that time, they have got high BP, they have got an emergency, yah there are times when we have got an emergency drug, we will give that client maybe lazex, but there are times when they will need maybe hydralazen. So a clinician would provide the thing to that client, will document in the book to say this client has got a high BP, the repeat was like this and it will be managed he will write all the plan. So that when the client goes to casualty, they don’t go there again to go and be seen again by a Doctor or clinician they just go straight to be managed, that is what we do. So in an event for example the client has come today to OPD, and they have their ART drugs, maybe their BP is just high even their BP drugs is there, so this client has come to be managed for the high BP but they have the drugs. So there is no way a clinician or a Nurse from OPD will say that because you are in ART go and be managed in ART, but I think when even when we are having a training, or this information needs to be disseminated I think that point should be made clear. We are not going to disadvantage client that because you are ART no. if the client goes to OPD, let OPD manage in a point whereby that client doesn’t have drugs, they have manage they finish they even do the info ART, then they just get this client and say, go to the pharmacy and collect these drugs and go home, that’s the whole essence of this program.

**I: Thank you very much, sister Penda, we have a champion already.**

R: Yes I can see.

R: So I think with me, I just wanted to make a comment, on the ahm, the in terms of ART, I just wanted to comment on triad. When clients come, are we just going to look at those clients that come for example, when they come you check the BP is high, they are the ones that you are considering, or all clients, that are hypertensive, like at that point when they come, the BP is just okay? Maybe I should, I don’t know.

**I: So our interest is all those that have both conditions, so you don’t have to have raised BP but you have to have both conditions, then we are managing you holistically.**

R: If that is the case, I think at the entry point at the triad, we should also just have a component where we will be asking the clients are you hypertensive, do you have sugar, so that you tick, you are able to verify them from there and you supply like that. Then in addition also, you know we are talking about the management of NDCs but I think some of us we have been working from ART for sometime. We will need more training in terms of management, maybe we forgot ARVs in our, so it is very important, that we have maybe more trainings.

**I: So just to mention that we will be doing point of care at testing so clients that will come, we will be there to ensure that if they are interested in being part of the study, we are testing them for all these different NCDs. Sister Cecilia I will come to you last.**

R: Okay, my, mine is on the entry points, sometimes, there are those who have been already screened. Some of those maybe they have BP or maybe they have sugar, for example the way we do, with DSD and main ART, even different guide, or maybe there is a tag. So maybe we put a tag, to know that this one has got BP or sugar, or maybe in the process, that person after being screened, they go to the screening room, they know that this person it’s when it is coming, they just put a tag. So that on the entry point we don’t ask the same thing, for example the way we are doing for COVID. Were you vaccinated, we write yes, again, the same question, we are repeating the same question, so if there is a tag, you need to help us.

**I: So we need to be able to differentiate our recipients of care, okay thank you very much. (Pause) no comment.**

**I: No, no, no we are not taking no comments, even just to say what someone has said, what has interested you the most? (Pause)**

R: Okay uhmm

**I: What role are you going to take?**

R: Okay since I am a treatment supporter, we are going to sensitize and talk to the people more often, so that they understand give more information.

**I: Okay, thank you very much, so we already have people willing to do the work.**

**I: (Unclear speech) Yes**

R: On my part, I will go to OPD, as I am a CHW, so as we escort clients at OPD, sometimes the treatment they give us, it’s not good, so we will need, your people to (group laugh) to be welcoming us well.

**I: Okay, alright, noted. Macdonald, you have heard, you need to tell these people to be kind to us as we support them.**

R: Yes, for me my concern I think is just to add up on what she has said, I think as we are introducing this integration, that will be the keypoint I might be good and she is complaining, this other person, it is their nature, they will be mean. So those people are there and those people will always be there, so as we are introducing this program, it would be very, very important to actually orient people on point of conduct. Me on that point that I suggested, I seriously see people in OPD and casualty say ART has introduced they are managing, just go to ART and I foresee, discriminated if we don’t do it properly. So I think there has to be a smooth introduction of that program and people need to be orientated to say at this point yes you can send to ART maybe for this service. Because I know it wont just start safe full casualty or full OPD and ART maybe that is my main interest.

**I: Sister Penda you have noted huh?**

**I: Yah**

**I: Yah, there is quite a lot I think that we need to do, lets start together at the end of the day.**

R: So for pharmacy I think orientation is best, not just for pharmacy but for everyone has to be involved, even the people that are not found in ART, they will have to be aware of this program, how it is running, how it is taken I think that is important. Then coming to commodities, because I know the commodities then this program is as good as being dead. So I think we need to sit down together, draw up a program and see how best we can move with this program. Where we have challenges we will be coming back to this table, getting advise from each other, seeing the best way to do it. I think I want to end by saying, I think this program is okay, it’s well accepted, I think I receive it wholeheartedly and I think when we are starting up a new program it always comes with a lot of challenges but I think those challenges will be dealt with as we are going we should be able to come back in this sitting and iron out our differences.

**I: I am happy that we are already engaging with our pharmacist also, I am sure the discussions are already ongoing.**

R: Yes, just like everyone has already said everything, so I don’t know, it’s, I am thinking maybe they can bring, as for us inpatient maybe they can bring part of, maybe I don’t know if it is a Nurse from ART, to be like specialized in in-patients, you find that we have got patients who are on ART and they have been discharged and they told to wait for the Nurse to come from there. And if you delay you find that they go, you tell them go to ART, they will just go.

R: Uhmm there is already a Nurse from is it (unclear speech) Patricia, by the name of Patricia.

R: Okay, like for now she is on leave so we don’t have any.

R: Okay, they didn’t communicate to us so we don’t have any,.

**I: Okay, it is a good thing when you have a boss, there are questions, there are solutions provided, quickly, alright thank you very much.**

**I: And just to add on what cristol was saying, whenever a program comes in, let us take interest to learn, we would learn, just as we are saying, we have been able to. We want whereby certain ahmm, certain activities, or certain duties which Doctor perform. Certain duties the lab person performs, they should be task shifted whereby even Margret will be able to do that simple task which they are able to carry on. They should be able to do that. So similarly with ART if you look at the guidelines, it says all Nurses, cause even right now into the general training for Nurses, ART is a huge component now, When we were being trained, I am an old nurse, it was greek, when you hear of ART, we couldn’t even pronounce it. So we could, I remember we could just say all those which end with Via they are drugs for, (Group laugh) so we take interest and the times we can even approach the in-charge to say can we have an in-house orientation on ART within the department and organize. She has a very capable team, sister Cecilia in ART, who would just come on the ward and orient each and everyone, such that you are not really (unclear speech). Such that the clients or the patients once they see a white uniform they have a lot of confidence in us, but when we say, wait for sister Getrude to come, or wait for sister this to come, they will say that one. In the community, you see people when they come they start discussing themselves from you, why because you just heard, wait for, so we show to the client like, you tell them the basics and say, okay I am going to collect drugs for you from ART. There then you will go, sister I have got this case, the sister will come and then it will be done like that, so it is something even in OPD we can do, it is not only in ART. And even when we come with the orientation we will want to orient all departments with continuous mentorship. So that every part, every person who is seeing the client, they will have this information and knowledge.**

**I: Ahmm, did you want to give a comment.**

R: Pardon?

**I: Did you want to talk about?**

R: Uhmm, I think for the pharmacy part again, maybe we can try to squeeze in other medicines like pain killers as well, cause mostly when the client comes that side they complain I have got a headache, then you, you tell them to go to OPD and they will be like just panado I got and queue up? So I think even pain killers.

**I: (Unclear speech)**

R: (Group mammer)

**I: Okay, so I think before sister Cecilia gives her comments and closing remarks, just on the intervention.**

**I: No we have one more women in defense**

**I: Oh, I almost forgot.**

R: And Moses hasn’t spoken.

R: I can speak after.

**I: She says she will speak after Moses speaks.**

R: Okay aside from the increased workload I feel managing a client holistically is better clinically, because you have a better picture of why the patient is taking this and that drug you know the possible side effects, drugs you can use. Unlike a case where a case is seen in ART, then they come maybe with an OPD complaint, they go to OPD and OPD is busy it is crowded, you find that even someone who is managing there doesn’t manage in full because they have not disclosed about the parts, the ART part, so overall I think managing clients under, clients with NCDs under ART is better.

**I: Okay, alright, thank you very much, so I will come to you finally.**

R: Okay, I think I will talk on the part of storage of medications, I think on departments like in DSD, they don’t have storage for some of the departments like for insulin and some of these diabetic drugs, I think that one can be looked into.

**I: Okay, thank you very much**

**I: Okay, you see, that was a very nice comment, which everyone overlooked eyy?**

**I: So before sister Cecilia as the boss gives a comment, I just want to thank you ah, for your time, you know its not. You know it is rare that we can get providers who are busy like yourselves and get to listen to some of the things that we had to say. So thank you once again, I mean we have learned for people like myself, the idea is everyday we interact we get to learn things that we did not know and at the end of the day we want to provide, uhm the best care we can to recipients of care because we know, these are our brothers, sisters, our parents and even ourselves as well. So if we are talking about providing holistic care to someone, I think it could be my own sister, my own brother, or myself as well, who is receiving care maybe not from here but from somewhere else. So we need to think about these things like that as well, so I think thank you to the team, for your time, and uhm you are going to be seeing more of us and we hope that you will welcome us with open alms. And are willing to share with us the knowledge that you have gained overtime and the experience that maybe one day I can say, he is a Doctor. (group laugh) so thank you very much, sister Cecilia over to you.**

R: yes ahmm, before I go to the conclusion remarks, there was a comment that, I mean a question about ahmm, creating space from that preferable which you haven’t told us anything

**I: Okay ahm, so that one I will refer it to the higher authorities to the bosses, I think you will meet them as they come, as we begun then you may interact with them, but it is something I will take, ahm forward as well. Then I think they will pick up the conversation with you.**

R: Okay, alright thank you, so coming to my concluding remarks this program it is a very good program of which way back, not even way back even today, we normally have these client with NCDs, most of the time the clinician will just prescribe and send them to OPD to collect some drugs. Of which I am looking at it, maybe a patient ehm, they are being inconvinieced by telling them go to the main facility, now since this program has come, at least it will be helpful, to help the patient to access everything at one point not like going and there is a bit of distance. You need to go round to go to the main pharmacy now one thing at the point of care, it will be good for them. uUhmm, I would like also to thank everyone who has come for this meeting thank you so much, I think we will us as Chilenje we believe in teamwork, we don’t work in isolation like ART we are there just on our own no. we go to the pharmacy interact with Mrs Hachinzwa Macdonald, everyone, we work as a team, so even this program will be successful, cause there is teamwork here t Chilenje and we believe in that thank you.

R: Group Clapping.

**I: Alright thank you very much, so I think those are the remarks I will take as well to my superiors as well, the key message we have take from here is that we are going to work together as a team so and that we are very much welcome here at Chilenje level one hospital.**

R: Yes

**I: Thank you very much**

**END OF INTERVIEW**
