# Supplementary material for: Applying human-centered design to adapt a multifaceted implementation strategy for integrating HIV and NCD services in Lusaka, Zambia: Healthcare worker perspectives
Source: PLOS Glob Public Health. 2026 Feb 2;6(2):e0005879. doi: 10.1371/journal.pgph.0005879 (PMC12863476; doi:10.1371/journal.pgph.0005879)
Supplement: S3 File — (DOCX) [file pgph.0005879.s003.docx]

**Interview Date: 24^th^ January 2022**

**No of participants:**

**Site: George compound.**

**Interviewee category:**

**Interviewer: Tulani F.L Matenga**

**Transcriptionist: Tulani F.L Matenga**

**Time:**

**I: So quiet (Unclear speech) is it true the findings we had during our discussion, was discussed, is it true the interventions are, are, are, what is presented, is it what is presented on the ground?**

R: Uhum [/yes/]

**I: Okay, why do you say so, why do you say yes we want to understand the meaning, when you say yes, when you say no. What is making you say yes, cause when we sat down, you all supported, these different ideas, that we, we threw to you.**

**I: So some challenges were highlighted.**

**I: Exactly yes**

**I: Because we might have missed 1 or 2 challenges or is it uhmm, implementation strategies which came out from the team, so that is what we.**

**I: Anyone feel free you can just talk, yes ma’am**

R: Yes so I can say it is yes because I have seen uhmm, a lot of clients when they come to ART and they have got other conditions like if we have got NCD patients and they are on ART they are referred back to main OPD. Equally OPD they see a client who is presenting the NCDs, when they see that they are on ART, they equally refer them back to ART, you are ART, go back to ART. So there is that, so it is not a one stop.

**I: So you are saying people are moving between.**

R: (Unclear speech)

I: **Which means that someone like for example if I come, I might end up spending the whole day right? Moving from there to there just trying to get care okay anyone else? Or maybe if I can ask a question, so we are aware that some facilities in Lusaka provide, they are already integrated, while some are not integrated. So for those are not integrated for example this facility is not integrated they are not providing care ahm, in this manner, what are some of the barriers and challenges, what are some of the problems or challenges that you face in trying to provide care in integrated manner? What are some of the barriers that are standing in the way of you providing care in an integrated way, or providing care in a holistic way? So what we are proposing providing care holistically right?**

R: Uhum

**I: So this patient comes, you don’t do your part and then exchange over but your are providing care holistically, you have got HIV I deal with your HIV, I give you your medications at NCD I also look at your sugar, if there is need for medication and anything else. So what is standing in, what are some of the barriers you are facing as health care providers? Yes.**

R: The biggest challenge is lack of (Unclear speech)

**I: Lack of what?**

R: Lack of commodities. Quite alright we can provide from our department but because we don’t have, we usually send them that side. So in the same way we have those, we also miss (unclear speech) will just take because we don’t have (unclear speech).

**I: Okay, so one there is a lack of testing kits for you to test recipients of care when they come?**

R: Yes

**I: Alright thank you very much, any other barriers that you face?**

R: Ahm, for those who are like critically ill, they have got other conditions so ahm, hypertension we will need to examine but space, space where to observe them but space is not there. (Unclear speech)

**I: Okay, so there is also a lack of space itself, what about provider attitudes themselves, as providers? Yes ma’am.**

R: Uhm, I think the other thing for this for it to be implemented, for it to work like effectively, I think we can have a day (unclear speech) where we have clients, though in Zambia, for example lets say Tuesday (Unclear speech) those clients who are on ART and (Unclear speech).

**I: Okay**

R: Why I am saying, because I am seeing in ART you find that a number of patients have come through and for us to be effectively screen everyone, provide the right care at that particular time ahm it wont be effective. No wonder we are saying that we send them that side because we know that side they will be everything, I think that’s what suggestion (Unclear speech)

**I: Okay, so there is a suggestion there is that we pick a particular day when you see recipients of care that have got both conditions right any comments on that?**

R: The problem is that everyday someone will come with a different condition, maybe its for the first time or they have just come to just they are those, you look at today it is high, they have just come the BP is high today, they are not, so you can make a day, but you need those other days, maybe their sugar will be high or this condition will be there (unclear speech) because they can just come anytime.

**I: Okay, yes.**

R: The reason why I have said that you should pick a day for them to come, it’s ahm NCD it’s not always that they are (unclear speech) we know that Tuesday is NCD day. These other days if they have problems they can come, even these other days for diagnosing you can do that, but just one day to know that today is NCD day so that (unclear speech).

**I: Any suggestion, I think there is a suggestion there and also my brother has also said that people are coming any day, so we need to think about that as well, any other thoughts around that? Yes.**

R: So, another concern is that, are you going to provide us with the Specific care pack?

**I: Specific**

R: Specific care park just to show the NHCs Profile per patient, because the ART has specifically will that be provided?

**I: Okay (Unclear speech, whispers)**

**I: (Chuckles) Okay, so uhmm, the whole idea behind is to, we are looking at this client whose HIV positive and has got either any of the NCDs, either Hypertension or Diabetes. And this, this is the same, basically we have seen that uhmm, Zambia, in Zambia we are focusing much on infectious diseases the NCD have been a bit neglected. And hence because we have resources under the infectious disease control and**

R: (Unclear speech, loud cough)

**I: Package of that support we have under the infectious disease support, so we want to leverage or we ride on the already existing pack and if we are able to ride on the already existing platform then we can have more support towards it. So as we have the ART care pack, when one, we find one whose hypertensive, we can just indicate high blood pressure or DM, it will, so that we don’t have a lot of documents. Again if we provide then with a lot of documents it will get back to us to say, ahh, there is too much to write down. But this is the same person and that is why even with SMARTCARE we just want to include that it is SMARTCARE as well, so that we don’t bring out a whole new thing, but within the existing platform, lets see how best we can integrate these services and modify so that it is able to carter for the NCDs as well, so much that even when we have this support it will just be an addition on the existing. Getting back to what our colleague said, we should have a day, when we started ART we used to have a day** isn’t it? What used to happen, ART day? Do we remember when we started ART?

R: People would come at anytime of the day.

**I: But again we were being overwhelmed, what would be the result of the care we were giving? It was like limited, cause we had a lot of people on drug ART, even up to this time we would have found all this place filled up with clients. But as we wait on evolving, evolving our medicines dynamics, we can do it, let’s integrate, let’s see if we can have it everyday. Let’s have a social market, we are having even a time, where, this is only 15:00 we don’t have any clients, we have a once off client coming through. So we want just to have that same thing because if I am , ahmm HIV positive I come in, with my HIV, then I have come for HIV treatment, then I should come back again, again on Wednesday because that’s when we have NCD clinic, it really has a bearing on the client, like they are coming twice. Even me whose seeing, I have already seen this person, I have examined this person, so we want everything to be under one roof, I don’t know how others would react to it. But we have seen how it has worked we had a one day ART day but as we went on with the system, it went on improving and now we have an open everyday we have ART and people are spread about.**

R: Just to clarify I didn’t say that, that day will just be specifically for NCD.

I: Yah I know, I understand that, that is what used to happen for ART, it could be we have a day for ART but even these other services are being handled, that is what it means isn’t it? It doesn’t mean that when we have the NCD day then the ART will not, the ART continues as usual, yah. Yes Ayuah, you had something to say?

R: (Sighs)

**I: Okay, that’s, it’s a really productive thing, we can do it for a start, we know how we will go about it, lets see what is workable for us on ground. We are the people who are doing everything. If I am here and I see to it to say, if we have this, we want to really see and put our house in order maybe we cant just start a supper market right now, maybe we will start, what is workable for us, that is what we had hoped for in this facility. What is workable in Chilenje is not going to be workable in George, so we adopt what is workable, what will, what will be beneficial to the community as well as the provider. Because if the provider is not comfortable with that it’s like a quality service, not at all, if I am not comfortable working like that then I will not provide a quality service hence the community will not be comfortable with it. So we want what is workable for the system in George.**

**I: Alright, thank you very much, one of the points I am picking up from sister Penda is also when she mentioned, what record, SMARTCARE so that is, that means, if a provider and you have this clients records, you are finding all the treatment history, in that record. So even their NCD management is also in there, so at least, you will know all the history and ahm anything else that you need to know about the patient, whilst you are seeing them. So I think that’s the key, we are saying, One stop, one shop, one record right? Yes ma’am**

R: On point number 3, there is ahmm, explaining and testing at the point of care, (unclear speech) so my concern is on point number 3, data collection should be manual (unclear speech) so now my concern is do we have any backup data collection tools or we will be using the same registers that we have to show that okay, on this particular day we saw NCDs. We saw 69 clients and out of 69 clients, 9 will have NCDs, do we have that? And then it’s SMARTCARE, when is it going to be integrated because last time I remember we were collecting the baseline data collection we couldn’t find any, so that is my concern.

**I: Thank you so much, so this study actually, has come in so that, to ensure that we have that okay. So we are piloting this study, so that we see, so as we start, cause, we are coming in on Monday just to kick start now the program, we will come with some ART (unclear speech) registers and ahmm, SMARTCARE, the SMARTCARE team, they have putched in some NCD component into SMARTCARE. Which uhm, providers, us as providers will be trained, when that time comes, we will be trained in that. So that we, when we click a button we will have that information, coming in, from SMARTCARE, we looked at SMARTCARE, we saw the gaps which are in SMARTCARE and ahmm put in ahm, we are putting some sort of solutions to pitch up those gaps which exist in SMARTCARE. Even as we come we are not saying that is exactly what it should be but even as we work, if I am 24/7 onto SMARTCARE, I would see the challenges, I would see how best, can this be done like this. Then we will pick it up and forcify (Unclear speech) SMARTCARE so that even as Ministry of health adopt and approve to say, yes this is workable, then we will all have a contribution, that is why we have come back to the team, so that we all have that contribution, Yes.**

R: Amm, since it is NCDs/HIV what will happen to those that only have NCDs concerning drugs? Are we (giggles0) are we going to be stopping, we are going to be stopping [/stocking/] ahm a variety of drugs here, now there is this client that will come just for the NCDs are we restricting just to give those NCDs/HIV?

**I: Okay, thank you so much, I will try my best to answer (laughs) my boss is here, my supervisor is here. So for ahm, for this pilot, we are focusing more on the HIV positive client, but for commodity we know that we have ahmm, the pharmacy. Yah, (Laughs) right, we have seen the challenges we have had with NCD drugs, so as we bring about this, it started with a higher level, Ministry tor those technical working groups they have been engaged already, we have go people who are dealing with them and they have reached the level whereby they have acknowledged that we have got a shortfall on the NCD commodities and Ministry has promised that they are working on it and still the people who are working with Ministry of health, they are saying, they are pushing so that they can procure more drugs for NCDs so much so that, we do not only focus on the study aspect but also to carter for the general population. Meaning even those that are not HIV positive but they have got NCDs too they will be (unclear speech) in one way or the other. In that, as a study we are not the one to procure the drugs so to say, we are only doing a small percentage just as backup but we are pushing Ministry to say, do, we need this, as we get onto numbers, cause we do not have these commodities at hand and most of the times we do not really keep records of how many hypertensive clients do we have for example? It is easy for us to produce, we will produce less numbers or we will produce inverted numbers and hence when they buy a lot of drugs, they will go to waste isn’t it? So we want to show that, this study is not that also influence the policy makers so that they will even calculate and procure drugs at least closer to the truth. So it will eventually tuckle for the non HIV clients who are living with NCDs, because it’s through the ministry of health.**

R: Then I might, another question, in case where we would run out of drugs, us as George we cannot place an emergency order, because we are facility edition, we are not a central edition, so what would happen in an instance where we would want to place an incoming emergency order?

**I: So as facility you order through Matero first level?**

R: Yes

**I: Is it first level or general hospital?**

R: General hospital

**I: Okay, you purchase through, I mean you request through,**

R: Yes (Cross talk)

**I: So ahm, we have a pharmacy person, a pharmacy liason officer who is handling the pharmacy component very well, it’s not my expertise, it’s my expertise as a Nurse. So he is handling that and discussions have, they have engaged in discussion even the emergency order. But for this study because it is only here in George and Chilenje general hospital, so ahmm, (unclear speech) had said, for the study commodities will stop at a bigger hospital which is Chilenje and then George will be ordering from Chilenje the study commodities that is. So once we have the study commodities we will stock them, they will be stocked in here, do we have space, to stock all the drugs?**

R: We don’t have.

R: We don’t have.

**I: Okay, yah, so bulky, bulky stock I think will be kept in Chilenje but then we have a, I am sure our able bodied pharmacy person whose leading the pharmacy team, will come and have a discussion and give way on how the logistics will be done at pharmacy level. as for insider departmental and the like, they help can help, I know logistics, yes as a Nurse will do the other because I also (unclear speech) drugs. Then you, just come to me then you, it’s to find where, is that okay?**

R: Yes

**I: Any other?**

**I: There’s a question**.

R: Okay, we are having a challenge of the BP machine

**I: Come again?**

R: We are having challenges with BP Machine, so if you can help us with 2 BP machine, then also the glucoseeds.

**I: We have the machine?**

R: Yes we have the machine.

**I: Which we charge K10? K20 now I hear, okay so for the glucometers and the BP machines, because this study is all targeting on NCDs and it’s conditions basically, so we have come in, as a study, we have come in with BP Machines, as well as glucometers and the sticks. Throughout, cause it will take a year, from, from today, cause today we are here until our year in September, 30^th^ September, we will be together so those commodities will be there for us. But in our mind, we bare in mind to say, we started with HIV positive clients, that is the one we are targeting. But sometimes, because we are in this facility, we will have an emergency and this is why (unclear speech) doesn’t have anything (unclear speech)**

R: The other thing is we also have a few benches, you can help us with some benches, you have noticed to say our clients they just stand, if you can just help us with the benches.

**I: You are truly sister Banda’s child! (Laughs) Actually that was noted even sister Banda our overall in-charge mentioned to say, if we could have ahmm, my supervisors when they came to pay a courtesy call, they (unclear speech) thank you for echoing that.**

R: Okay

**I: Yes.**

R: Is mental health also included?

**I: That is a good point, okay so mental health is under the non-communicable diseases and for us we are focusing on these 3 conditions but when we find a mental health condition or we find a client with mental health, we should have linkages. Where we will link them and ensure that they get appropriate services for that, but for this study, why we focused on this study, we want to see that later on eventually, once Ministry of health sees the result as we work together here and produce the result, Ministry of health, will now strengthen so that the people in mental health and other conditions which we are not currently focusing on, they are also encompassed, any other?**

**I: So I like what sister Penda said, in terms of, so the key message really is, we will be here as a study supporting you with medication, testing kits, training and other things that are, in order to ensure that we are providing best of care to our recipients who are living with HIV, the idea is that we want to provide evidence for Ministry of health, when we are here we will be piloting. So we are going to be piloting 2 facilities, Chilenje and George, when we do our pilot for a couple of months, then we have results right? Then you tell Ministry of health, this is what we have found from George and Chilenje then at some point we roll out other 15 facilities. Then we come back and say Ministry of health look, this is how we should be providing care to people that are living with HIV and NCDs, it should be integrated at the end of the day. You can’t provide care separately but let’s do it in an integrated approach, then now we hope that Ministry of health will take it up as their own child and this is how things should be happening when one goes the facilities. So this is what, this is the evidence we are trying to come up with, in the future we want this to be happening at the health facility. So this brings me to my next question, how do we ensure that this integration that we are talking about is accessible or it is being practiced in the health facilities when we come here and say this is how things should be done. How do we ensure that it is going to be happening, cause we can just be saying integrated right? We say oh George is integrated, it is an integrated facility, but integration is not happening, I come, HIV, I get my medication everything is done here, go to the other side so we are saying integration, but it is not happening, so how do we ensure that integration will sit with, it will be happening some recipients of care will know about it and will be accessing this package and will be attended to holistically? I just want to hear from you, how this is going to happen, yes.**

R: The first point is availability of (unclear speech) that’s the number one.

**I: The number 1, okay, so you are saying if we provide drugs and if we strengthen the system of having these drugs and they are available then clients will be seen holistically?**

R: Yes, I think training of (unclear speech) so that they keep on.

**I: Training.**

R: I think also training for us clinicians, because let’s say for example, here I am I am a Nurse I have been working from ART since, let’s say 2008, yes, it is very clear that you need to work with a client with NCDs such as hypertensive, DM and these other conditions, in such training is also a vital part of the same study.

**I: Okay so we need to provide training to the providers?**

R: Yes

R: Yes

**I: Yes ma’am**

R: Okay, on my part I think community sensitization is also important, as a client when I know to say if I get ARVs from ART I am diabetic, I need to be seen as well, I need to be seen, so when they know the conditions are not supposed to be referred to then that will help us. Infact they can even remind us to say No, you remember last time you told me that even this condition can be seen from this department. On that one then you are going to make sure that we do the (cross talk)

**I: Okay, just on the community sensitization, eh, just to let you know that we have already started engaging the community. Yesterday we had a meeting we had a meeting with the neighborhood health committee from George and Chilenje so on that one we have already started engaging the community, so that they spread the message in the community, so that one is also something that can be taken care of.**

R: Okay, I feel it can be also something that can be done by the drama group sensitization, the way we used to do it when we just started giving ARVs from the facilities. People were shunning to come to the facilities but we had to take the ahmm them right in the community, do performance for drama and then from there we even give some people who have got those conditions an example to say like me I can get the services from the same (unclear speech). Drama sensitization right in the community can really help us.

**I: Actually those activities are already line up, what has delayed is that we had these Covid, so we avoided a lot of things, gatherings but now numbers are going down that is why yesterday we started recruiting the NHCs. So we are, we have lined up a number of community sensitization including the churches. Like for Chilenje before the Covid numbers started going low, we actually visited about 3 churches just to sensitize the community. So even here we will come, it is just now that we are starting.**

R: I think on sensitization, let us also include, like when clients come with their previous documentation (unclear speech) when they come for their diagnosis. Because if we leave that out, they will say if I go that side I will be given the drugs but we should also remind them that come with the right documentation.

**I: So you want their treatment history right, so that you know the other information on other drugs and everything else?**

R: Yes

**I: Alright, so this is uncle Peter he is an expert in community things, like I said we are a small group but large, in that we have our specific fields I which we are covering and which we are here to support you. So community, issues Uncle Peter will be here to support you, Dr Telo will support you in terms of training, we have Brain our pharmacist who will support the drug procurement system, we have Chilambwe whose going to be dealing with the Lab and you have me whose going to be here just to observe talk to you once in a while. My goal at the end of the day is to understand some of the challenges and how we can push the agenda forward, any other? I think we are talking about how do we ensure that integrations happen at the facility? Apart from providing drugs, ensuring that the community are aware, providing the testing kits. What else do we do to ensure that integration is being practiced when people come to the facility? (Pause) Oh maybe I should ask, how do we sustain it? Because we will be here for a couple of years then we are going to go as a study and then we need to say now this is the evidence so it works, let’s integrate it, so how do we ensure that it is sustained, it goes beyond our time as a study?**

R: Uhmmm (sighs)

**I: I want to hear your suggestion as George, we will be here to support you, drugs we will be here to support you, training, mentorship, providing information, provide medication, but a time will come for us to go, when the funds from the funders are finished right? Then we pack our bags and we go and then we say George this is your baby now, so how do we ensure that it is going to go beyond our time here as a George.**

R: I think you have already answered that question, why I have said you have already answered that question is because you are saying that as a study you have come to provide drugs, you are going to provide mentorship. If that will even be the ministry we will continue (unclear speech) but I think we will sustain it.

**I: Okay**

R: Yes.

**I: Okay, so you are saying once Ministry is involved and takes over, it is going to be sustained.**

R: Yes

**I: Okay any other suggestion apart from the answer I have mentioned, any other? So once, we want to hear from you because this is going to be your, you are once provide this evidence George is going to be the example or you are (Coughs) this facility, this is where we have done this. If it works or it doesn’t work, remember we are trying to provide evidence, so if it doesn’t work we are going to say it doesn’t work because it didn’t work at George right? If it works it will be, it works because it worked at George, so we want as many suggestions from you, or maybe I should ask, if ahm, this is what we think as a research team. But if we threw it to you and say how would you manage people that are living with both conditions, what could you be doing? Or what kind of intervention would you design for them? What would be your take, or among these 5 things that we are proposing right, this is our 5 proposal, if we gave it to you, what would you take our from here and what would you add? To say that one cant work here at George, me I would rather do this, for example us we are saying one stop, one shop, we are saying ahmm, OPD for NCD management should come in the ART clinic, that is our proposal, someone else can say no, why don’t we have a different building where we are going to be seeing (Loud cough) living with NCDs. Just like we have ART now, people who are living with HIV don’t come there, they come straight here, you say okay in the corner if you have both conditions you are going to be coming there, so I want, those are suggestions I want to hear from you. (Long pause) what will not work here or everything is perfect.**

R: No number 3

**I: Number 3 provide access to cater metabolical conditions screening and testing using point of care diagnostic tools in the clinic.**

R: (Unclear speech) so for example, (unclear speech) type of condition, to (unclear speech) facility. We want ahmm, what is there in like under the SMARTCARE how do we receive info, of how that man was cared for and (unclear speech) Are you going to follow it up so that these plans when you are done that side and they are referred back to us, is there I don’t know how I can put it maybe, I don’t know, I don’t know.

**I: So you are trying to say, if anyone is referred or we can’t provide the service here right, they go somewhere else, is there a way for you to receive feedback in terms of how they are treated?**

R: Yes, and (Phone Vibrates)

**I: Yah I think the secret lies in that record cause our hope is that if anyone receives care, anything that has happened should be in the system, should be on that record, so that for example if I come back to you today right? It means that I have all this history. If I have been in this facility, I received this drug, it should flag up or you should know okay, this person went to this facility and this is how they were treated for example, so the secret I think lies in the record itself and that is why data management team work on ensuring that they strengthen SMARTCARE so that it’s able to show you as providers, this information as well, any other ah. So one of the points as well I thought we could talk about is the first one, how might we integrate the TASKPEN package into ART clinic work flows not to overburden health care workers and make patients wait too long in the facility. Remember what I said is that one of concerns we raised is that you are going to overburden us, we are managing ART already and now NCDs. So it means when a client comes we manage HIV and now you have added NCDs so you said you are going to burden us, but what we are trying to find out is how do we ensure that we don’t overburden you and make patients wait longer? We want suggestions from you because you are the ones who are going to be doing the work, so you need to tell us, how do we not overburden you with so much work. In addition to already what you are doing then we are adding on a task? Yes!**

R: (unclear speech) is if we have enough commodities.

**I: Enough what?**

R: Enough health commodities whereby we have enough drugs whereby you don’t have to you don’t have to go around looking for it.

**I: So what I am hearing is if we provide these things, then it is going to work and it will not overburden you.**

R: Yes

**I: It will work, alright, okay and then the second one is, how might we encourage treatment supporters and community health care workers to take on the task of educating people living with HIV on NCDs and tracing patients who miss NCD medication? So in this case what we are asking is, in terms of the treatment supporters how do we encourage them ah [21:08-20:48 Inaudible segment] Yes (long pause) Yes**

R: I think for point number 2 it is just what we always do, maybe discussion in the morning before work (unclear speech) updating each other, helping each other, we give them more information that can help the whole team to do what they need to do concerning the client.

**I: Okay alright and then maybe a second follow up could be.**

R: I just want to, on point number 1 on the issue of overburden, so for us health care workers I am agreeing, I believe now, apart from (unclear speech) and also In charge and maybe there is need of something maybe sugar test or something and also someone maybe, (unclear speech) someone who can be taking it up. Cause maybe I for instance I need to take someone, I don’t know to say in fact I will just stand, I will go to the lab, I think there should be that communication if our client, I think that will help.

**I: So ahm, sort of developing a communication system right within the facility so that you are able to refer if you know who is supposed to perform the test and things like that**.

R: Yes

**I: Okay, alright, ahmm, so one of the things that we, I want to also hear your suggestion is someone somewhere, someone had mentioned that you need, for this particular integration to proceed in the future, you need to appoint champions and HIV ends with the champions who is going to spearhead this agenda, I just want to hear you, what you think about that particular approach. Should we appoint an HIV/NCD champion who is going to drive the agenda even after the study is done, someone who will ensure that integration is happening, people are receiving care holistically, should we appoint a particular individual who is going to be the champion of this particular management of care.**

R: Yes that point is very important.

**I: Why do you say it is very important?**

R: If, when a champion is available that person it’s like they will spearhead as a supervisor, so that one can work very well for the champions. But we are suggesting the champion should be a clinician.

**I: Champion should be the clinician?**

R: Yes.

**I: Okay, alright, any other suggestions and thoughts? And how do we ensure that TASKPEN is usable for facility stuff, so our approach is called TASKPEN in short so we want to find out, how do we make it accessible and usable for facility stuff? How do we make it usable for you and accessible or desirable? How do we ensure that when a client comes, you will want to treat that client holistically, what would make you?**

R: I thought that one it was already answered because you said did we have the commodities and the knowledge, definitely I would want to do that, yes and if also the community, they also have the information.

**I: Information**

R: Yes

**I: what about ahm, clients ahm providers attitudes, some of their beliefs, maybe there are those who believe you cant ahm me I have been trained only for HIV I cannot manage NCDs, those are attitudes. To think about such kind of provider attitude and maybe experiences that could maybe block us from providing care holistically? Are there providers who think that, me I have been trained, I can only manage NCDs things to do with HIV that cant happen, take me back to school for example. Want to understand in terms of provider attitudes, provider beliefs for example.**

R: If I said it would be negative cause before someone was trained to do the training, apparently they went to do, some of these NCDs, so I think just like I mentioned in the first place that a refresher course because, someone was coordinating a certain, it becomes now, because we have shared work. Someone has been doing HIV for a long period of time I think mentorship maybe for 3 days or given maybe brochures or materials to read. I think they can also embrace the same NCD care to the clients, so I think

**I: Mentorship and training will be there it is a must, otherwise we will not manage.**

R: Especially I need guidance on how to manage a hypertensive client and I am not aware on how to, I still have old one, old information how am I going to provide care which is holistic to the client? So mentorship and training is very important

**I: Okay any other comment? (long Pause) nothing? Okay and then maybe just before we conclude our discussion, so I was trying to understand I think we discussed in the facility, I cant mention going back where we begun (Unclear speech) so one of the things someone said, one of the providers said is we need to, for us to know that integration is happening is we need to have a reporting system, right? Monday is it every Monday we should also report on clients that are receiving care in an integrated manner, so what are you opinions in terms of that? For us to know that recipients, you need to have a reporting system, as you are reporting for other, the other things that you report I don’t know if it is on a monthly basis or a weekly basis are we still supposed to be reporting for example we say, we have seen this week, the past week we saw 10 patients who are living with HIV and NCDs for example and this is how we managed them.**

R: That one it’s a must I think there should be some sort of reporting so that we know what is happening on the ground.

**I: Okay, any other additions? Not much people are tired ey? (chuckles) so in concluding what I will do is, I don’t know who is the in-charge?**

R: Just a question, if you have to report, even if it’s a register or we have to provide our own books? How are we going to report?

**I: Okay so what, that is a very good question, so that book is going to come from us like as providers what do we suggest, what works, or what has worked in the past, cause remember us, we are coming in to support, so when we say providers should report, our job is to give you the registers so that you will be able to record. Yah so when you say you want to support in this manner, then we will support you to ensure that you have that system in place that you can use to do the reporting, yah. Suggestions then, since I am hearing different reporting system, can suggest to us and we take it down as this is how we should report HIV/NCD management.**

R: (Unclear speech)

**I: Nothing yet, since we will be interacting, this is not the first time, that you are seeing our faces, we are going to become best friend, here in offices, knocking, some of us are not providers, I am not a clinician I don’t have a background in medicine, I have a background in social, ahmm, in development studies and social sciences. So I will basically be sitting there trying to observe what is happening and questions as well. Here we have things that we want to talk about, we will come and talk about. The experts are also going to be here training and many of us as well. So this discussion will still go on, we will still come back and ask you is it working, what are the challenges tell us we go back and shift things here and there. So in concluding I am going to ask who is the in-charge? She is the in-charge, okay, so I am going to, I will start by giving the closing remarks myself, then uncle peter will say one or two words, then everyone is going to make a comment, they can comment about the presentation itself, you can also comment about how us as a study team, can work together with you cause remember we are not MOH stuff, sometimes we might, (unclear speech) they come to disturb how you are working, because they come give extra work, but then we are saying how do we work together we want, we don’t want to interrupt what you are doing, but we want to work together with you, we want you to depend on us for help, we also want to depend on you, so you can also talk about some of the ways in which we can work together and also to make a comment. So I am going to start by saying thank you very much for giving us this opportunity to learn from you, for us, when we speak like we are learning at the end of the day. So it is just to thank you for your time as well for giving us, we know you are tired and doing so many things, this one hour that we have sat I think we really appreciate for that. So I will hand over to Uncle peter and then I am going to go round the room then the In-charge will give the closing remarks as well.**

**I: Okay, all I can say is that thank you so much for our stay, you know we have come here, you have supported us, we have been here from last year, you were not seeing us I know, maybe we could have seen 1 or 2. But what it is, is that the support you have given us already should continue so that this thing can succeed then when it succeeds it is for the betterment of our future. (cross talk), thank you so much for your support from Monday next week we are here full time.**

**I: You can start from here**

R: I think we have, if this was to work out, whenever we bring, I am feeling for the pharmacy, when we request for something, the commodities and all the expenses (unclear speech) that you try and push in, so that we have a quick response, then we can.

**I: Okay, alright thank you very much**

R: Drug prescription again.

**I: Go on.**

R: No he is adding onto what I am saying

**I: Okay**

R: Drugs should be there, unlike we see them, they have to go and buy, sometimes they could have just gotten time.

**I: Yah, okay alright, thank you very much, you can start so that we can go all (whispers), yes my brother, you can, just give your concluding remarks, we just want to hear from you before we wind up.**

R: Just to, the only few points that I can just bring out is as we start this project, you just need to ensure ownership of the project, so that there is sustainability and everyone will be involved.

**I: Okay.**

R: But also, just emphasizing on the issue of commodities especially for the labs and also drugs that can be continuous supply, I think that can be a success. And also I am hoping more training so that our clients cant get to know more of this duo conditions, I think that is what I can say.

**I: Yes ma’am**

R: (Sighs) For me I think for this program to be a successful one, there is need for proper sensitization for those people who will be involved in the sensitization process, without sensitization the project cannot succeed. Because it is through sensitization that a lot of people will be aware of the program that will be underway at this facility, that’s al I can say, and also there is need for collaboration.

**I: Collaboration okay.**

R: We really need to work together not in isolation no.

**I: Yes ma’am**

R: You are welcome to George and we really need to kick start this program because we started expecting it last year, so we are happy that it will soon be here.

R: I am just adding on to what madam has said, I am madam Belinda working from MCH so I will talk of sensitization it is very important. Because in the morning we had a pregnant woman, whose on ART but again then temperature, the BP was high, so it is very, very, important for us that side, it will be easy for us than sending the patient to OPD can see the patient at (unclear speech)

**I: Alright, thank you, yes.**

R: For me I will still stand on the 2 points I discussed earlier, sustainability of commodities and also regular orientations or training, that can really help to push us to sustain this program.

**I: alright thank you very much**

R: Me I am going to say something that is not

**I: Okay no problem.**

R: I think if you were to do another study, or maybe after this the pilot works out and you roll out to 15 facilities, I think it is important that you should include mental health. Because I think we have observed one thing as our ministry mental health is being sidelined, trust me George we have a lot of mental health cases, but because we don’t have a department and someone whose trained to handle those cases what do we do, we send those clients somewhere and we are saying that we want a one stop shop. So you find that this client is mentally is on maybe drugs for mental health but he is not accessing them here. So I think you should make sure that if this works out and you are rolling it our to the 15 facilities, please include a section for mental health.

**I: Mental health (cross talk) alright, alright, thank you very much, yes ma’am**

R: I want to second on what my sister said, for me I can say, thank you so much for engaging the community and this community, this George we have 26 zones or 22 zones so all 22 zones, I think the information should reach in these zones, through sensitization.

**I: Okay thank you very much, yes ma’am.**

R: As for me I would like to thank you for this program, a lot of things have been said, I think it will work, so that all the other facilities it is going to work for them.

**I: Okay thank you very much, yes ma’am.**

R: As for me, I just want to appreciate for this project cause last time when we had the discussions what we, we used to present to you have even put them on that list, so I think even for our client it will be easy instead of them moving up and down they will be just getting the services at the same place, so for that I appreciate.

**I: Yes.**

R: Even me I appreciate for this program many people will be helped.

**I: Okay, that is all, thank you, yes ma’am.**

R: Yes for me, I just want to appreciate what you are bring here at George it means you have made our work simple.

**I: Okay, alright thank you very much, yes.**

R: As for me I appreciate on my own, cause at least a lot of clients will be helped and even other, it shouldn’t just end at George even other facilities they are facing the same challenge so at least if it can even matero south area so that everyone can access the same service.

**I: Okay, thank you very much, so sister Penda, you didn’t say anything, so I will ask you to say anything, then I will hand over to the boss to.**

**I: Uhmm, I don’t have much to say, we have had a very live discussion and we have really gotten a lot of input which is going to help even in the study, even as we come back we will be able to interact and it doesn’t mean we just have to stick to what we have thought in our head but we open so that we want this to work not only for the study but for the future, so we are open minded and to incorporate your ideas, so that we achieve the goal, so that ministry as well, we will help Ministry to put up policies as well as other guidelines which are, which are going to assist us in our executing of our duties into these facilities. I think, its been a great discussion, I really appreciate and I have gotten 3 things which will build on whatever I was thinking and also tune my thinking as well (laughs)**

**I: Alright thank you very much, sister over to you.**

R: Thank you very much for everyone who has come, I am sure most of you didn’t even go for lunch you were waiting patiently for just this meeting, we really appreciate and we are looking forward for this program to be implemented and also for the positive results. So if you face any challenges please, come out, let us solve those challenges together, and thank you so very much.

**I: Thank you very much sister**

R: (Group Clapping)
