# Supplementary material for: Applying human-centered design to adapt a multifaceted implementation strategy for integrating HIV and NCD services in Lusaka, Zambia: Healthcare worker perspectives
Source: PLOS Glob Public Health. 2026 Feb 2;6(2):e0005879. doi: 10.1371/journal.pgph.0005879 (PMC12863476; doi:10.1371/journal.pgph.0005879)
Supplement: S4 File — (DOCX) [file pgph.0005879.s004.docx]

**Interview Date: 26^th^ January 2023**

**No of participants:**

**Site: Mtendere**

**Interviewee category: FGD**

**Interviewer: Tulani F. L. Matenga**

**Transcriptionist: Tulani F.L Matenga**

**Time:**

**I: So anyone can feel free to give a comment, go ahead.**

R: if all the logistics required are available it wouldn’t be a burden, but it becomes a burden if the required logistics are not available.

**I: So you are saying you need to have logistics available?**

R: yes you need to have all the logistics available

**I: Aha**

R: you need logistics available in all centers of care so that clients can easily access them

**I: Accessible**

R: Yes if you are easily accessible then all is well but if you are not easily accessible now starting to explain saying no we don’t have or direct them to another facility or you need to buy then it becomes a burden.

**I: Okay, thank you very much any other comment? Yes pleas go ahead.**

R: The is that it not burdening to health workers, the burden comes in when you start thinking of where to take the patient if can do it at one stop then it’s not a burden.

**I: what I’m hearing is that, in terms of extra work it’s not a big challenge for you as providers okay, any other addition? We’ve heard about providing deferent drags that people need right, and also ensuring that they have these medicines at home, patients come we are able to do thing in a holistic approach yeah, so any other, mmm, I want to hear is there anything we can do to stop over burdening health care providers.**

R: As you have mentioned that treatment supporters also play a role that will be lightening the burden of the staffs because they will be able to receive clients, do the BP checkup do everything needed do sugar test, after their findings, they will now transfer the clients to the nurses or the clinician for the final findings yes because they are there to receive clients and to do the first aid the it will be not a burden for them because they will be receiving people with the needy rather than getting everyone then they start screening themselves it will be a burden because there are a lot of clients sometimes in ART clinic.

**I: so one point am getting is that in terms of work load this is not a problem everything is okay right Sister in charge there are pros just that as providers the experience has been if we say let’s manage this conditions together for example we say we are starting today, I know today it’s a bit of a smaller facility so there’s no lot of movements in terms of care, we say okay this is one stop one show for example this room where we are managing kind of care holistically where us the nurses community based health workers, treatment supporters are managing this from front comes one person and provide all the care holistically. So what I’m getting is that health care provider we are able to manage without being overburdened okay.**

R: unclear speech

**I: I’m hearing manpower maybe, yes sister.**

R: Manpower is there though there’s shortage of manpower but when it comes working culture hear at Mtendere health center we are able to manage reason being that we are working as a team there is that coordination of work and also there’s that division of work. The way you explained earlier where you talked about the counselors the community based health workers doing the sensitization and also linking the clients from the community to the health center. Definitely when they come here what we are going to do, we are going to receive them and also offer the services that are needed to them. So you would find that when they go to OPD there are people there at triage, which they are going to identify to say this person need services abcd so that is team work where we are going to link that patient from triage into the ART and ART they are going to offer their services. They will link that client to clinical officer also the pharmacist so we can’t complain that there is work overload division of work is the one which really motivate us and that team work it will motivate more when it comes to service delivery.

**I: Alright, thank you very much. So I’m hearing that there’s a system that you are using where there’s division of labor. So because of that system in existence makes things easier because people know what they are supposed to be doing at particular point and things like that.**

R: Yes

**I: Alright thank you very much. So the other thing I will ask is that how do we,, what we are discussing when we are in Lusaka is that some facilities are said to be integrated while some are not integrated. So I want to find out in terms of, what are the some of the challenges, what are the barriers to integration? What stops integration from happening for ACD HIV management what stops, because we are aware that one patient is the same individual that you are sending from me to the other service provider. So what are some of the barriers that stand in the way of us providers providing holistic care for this one individual?**

R: Actually for Mtendere it has been integrated isn’t, it has been integrated so the system is already there and also if there is maybe a surplus of work that is needed to be integrated for us it will be easy because we already know the system how it works, it is there and we know how we will do it.

**I: How you do it?**

R: Yes.

**I: Alright.**

R: Maybe an addition

**I: An addition**

R: Just the same thing, we are already integrated it’s just maybe the logistics that are not yet in place like test kits the drugs as long as everything is in place all is well.

**I: All is Well okay, so I’m hearing that this system is already hear right?**

R: Yeah

**I: Except maybe it is not working efficiently because you have these gaps in terms of patients and test kits. So one of the things I would like to find out from the people from pharmacy is that, people from Zambia Medicines and Medical supply ARGENCY [ZAMMSA] are saying that drugs are there Except that facilities do not request for these drugs so you find that there is shortage at these facilities. So I want to hear from you in terms of what challenge is there at the facility and as, I remember we had a small study right, hoping to influence the ministry to take approach as we head into the future but starting at very small level what can you do to support, is it that we need to change the procurement system or providing drugs or we need to provide these drugs from the pharmacy.**

R: (laughs) either way it works, but the thing is probably the supply chain.

**I: The supply Chain?**

R: Yes, that’s the one you need to work on.

**I: There’s a problem with the supply chain so we need to come and strengthen the supply chain?**

R: I think that the work for the people at ZAMMSA and us to work on.

**I: So the Problem is at ZAMSA?**

R: No I’m saying ZAMMSA and us because ZAMMSA has said it is us, we will also say it is ZAMMSA, so let’s just say the two of us.

**I: So the two of you will need to find the system of**

R: Workable for both of us

**I: Alright, thank you very much. So in terms of how do you procure the drugs?**

R: We don’t procure we just order

**I: You just order?**

R: Yes, so

**I: Is there a system that you are using to order the drugs**?

R: Yes.

**I: And how is it, is it user friendly I want to learn because I’m not the provider myself (laughs), I want to learn as much as I can, as a lay man?**

R: We use both the software and the hardcopies. From ZAMMSA we use the software where we report every month to send the report from which they provide the drugs for us. Then the hardcopy we go to Chilenje hub for sustainability of the drugs.

**I: And does this pass through the in charge or as pharmacy, there’s a chain that you have?**

R: As pharmacy yes.

**I: So the in charge is also aware what they are ordering, what is not in the facility, for example the need provider are they aware that this drug is there or not even as they write a prescription?**

R: Often time they do because we work together as one so they are usually aware of what we have and what we don’t have.

**I: Alright, thank you very much. I think we are trying as much as we can. As a team what do we do because remember for us as the facility we have different people, we have some in the pharmacy some in the lab. The idea is to provide the care ourselves know to the facility, to come on the ground and support health care providers using different system method, should we train people and things like that. That’s where we are looking at as coming in. Any comment any question? Yes**

R: As researchers, are going to provide drugs depend on the pharmacy chain as I have head the madam explaining about the relationship or maybe you people doing this program you are ready to bring drugs here even if we depend on pharmacy they cannot depend on modulate all the time, people take different drugs then pharmacy is supplied with modulate. It’s not everybody who takes modulative drugs. So as long as drugs are not found in pharmacy it is not going to work because we will start inviting people to be insulting pharmacy if we start this program it’s better we leave people the way are to go and buy the drugs not us telling them that now this is what is going to happen and yet it will not so it will be the lot of burden, the burden we are talking about now it will be worse.

**I: Thank very much, it is a very strong concern and I think when we here our seniors talk, they are talking about how important pharmacy is whole approach because if there no drugs then we can’t integrate because it means that we do all these thing diverted somewhere we cannot provide the care yeah. So one of the things we are saying is one is to provide from these drugs depending on interactions with the pharmacy so you find that in our group there is a pharmacist whose job is to rehearse with the pharmacy in terms of what is the problem and how do we assist through to order the drugs from ZAMMSA or you provide some drugs. So we have engaged ZAMMSA and they are saying that we have these drugs so people are not ordering, we have engaged ZAMMSA to say drugs are not at the facility and our study is looking at how do we provide care holistically without the drugs we can’t provide care so we have engaged them and the team is ready to supply some of the drugs are ready to work and also as the team we are ready to supply some of the medication once we go out to the facilities we are able to supply in terms of the basic drugs that you need because you know what, medicine stock at the clinic is quite a lot of, now you see people are coming to collect these drugs. Our hope is that if we are here also assisting providing some drugs and also ensuring that we support in terms of how we order these from ZAMMSA.**

R: I feel the ones that supply these drugs for ART drugs have the better supply system so if we want to integrate the best is if ZAMMSA can give that role to the hub that the ART have so that all the NCD drugs provided under the ART should be provided through the hub that provides the ART drugs and the supply chain will be better.

**I: Okay, so you are saying there’s HIV drug procurement system that is much better than OPD?**

R: I wouldn’t say the procurement system but the supply.

**I: But the supply, so that supply is great ART program.**

R: yes.

**I: Okay**

R: So maybe let those drug be supplied specifically for the ART let them be supplied under the ART.

**I: Under the ART okay. So one of the things I’m learning from you is the pharmacy have also these medication bring everything there so that when the time come they come and collect both just like I proposed right?**

R: Yes.

**I: Alright thank you very much. One of the things I want to hear, one of the challenges I think we have sister is that organizations come right like the way we have come with a specific project and treating people for some time for two three years and then at the end of project life we pack and go then now the program also die, so you start telling them that the people who used to provide HIV service are gone, which means we are going back to how we used to be right. So we are here as ART/OPD people are being manged here holistically, three years we had finished people are going back to ART/OPD so one of the things we are trying to say is, how can we ensure that we sustain this at a point when our team has finished and give the ministry of health the evidence that this works. How do you now as facilities continue with this particular approach, how do you sustain it, how do you make it your child or your baby at the end of the day once we are gone, once we are gone, once we are done supporting you, for the different way we are going to be supporting you for the couple of years, how does it become part of what you do? How does it become part of the team in health?**

R: The suggestion I made is one of them.

**I: Strengthening the**

R: give it to ART.

**I: ART**

R: To supply those drugs because that means it will be a continuous process even when you are gone, they will continue with it even when they know that you are gone, they be providing ART drug including NCD drugs.

**I: Okay, Alright thank very much, you had your hand?**

R: Yes, I think one of the things that make the program die is the there’s continued manpower coming and going, coming and going so maybe there should be continued orientation of the new staffs so that the program can continue and sustainable.

**I: So you are saying people are living almost on regular bases?**

R: maybe other suggestion could be if we appoint a focal point person where we are going hand over the whole project under maybe sub district level or district level to ensure that there is that supervision at the facilities so that there is that continuity of care maybe probably it can work in that way, beyond Mtendere so that it can be under sub district so that someone could handle that project so that they keep on monitoring.

**I: Okay, you are talking about focal person right?**

R: Yes.

**I: Some of the things also we hear is that Chilenge is having a champion So I’m looking at it that we have the similar situation where we have someone who is spearheading HIV AIDS integration so that even if we are not there tomorrow we still have this particular champion who is carrying the agenda to that this is happening, alright thank you very much. So we are speaking the similar language with other facilities right, any other contribution?**

R: Are these people who are not under the ART/NCD going to benefit from these drugs?

**I: Yeah (laughs).**

R: (Laughs) because they will come a situation where this patient is not under ART and they are prescribed this same drug and this patient under ART has been given this same drug, I don’t know if you understand.

**I: yeah, It would sound like discrimination.**

R: (group laughs) are they also going to be benefiting as well.

**I: so some of the reasons why we are focusing on people who are living with HIV are on ART is because evidenced has shown that are with ART are more likely face disease because you aware that the drug they taking itself one, is very strong, we are aware that ARVs are quite strong. Some of the drugs that would cause people to have Sugar to have issues to do with fats all those different thing, again we know that people that are on ART are living long, long time ago was a very scary thing right, but now people are living 20 to 30 years. So now what is important is, we are now moving toward quality of care holistically care for this individual right. Because we are know that you are living longer, you don’t worry about people dying anyhow but then can we give them the best care. So I see where that is coming from where you have that concern, of course as a facility we can’t be saying this medicine is for people living with HIV while at the facility we have people who not HIV positive but they need drugs as well. So of course these are some of the things that once have drugs we come together and say how do we incorporate people that don’t have HIV but they need drugs as well so there we don’t want to temper with the system at the facility, that’s not our aim but to come and say how do we do this but of course our interesting is to provide this care and also present them with attention, that should be our aim, I don’t know if I have answered the question but I get your concern, you know as stake holders are saying let stock drugs for for people living with HIV cause stigma sometime and not anyone else, they say we want to assist pharmacy in the way they order the drugs so that we are in between ZAMMSA and the facility, so it means that we are asking the facility how much NCD drugs they consume in a month so that even as we are procuring what are we procuring for that particular facility at the end of the day it’s the same facility right, I don’t know if I make some sense there? You can tell me if I’m not making sense.**

R: Group laughs.

**I: Okay, any other contribution? How do we make this integration accessible how do we make it user friendly so you as a provider, the key message here is that the provider provide this aid right. How do we call people to come and train us, can we manage these conditions without being trained?**

R: (Group laughs) we are hoping for that actually.

**I: For training right?**

R: Yes.

**I: to come and train you, providing power and training as well. So some of the team members are medical doctors and their role is basically to train providers. Myself and collective researcher, mine is to interact with you providers we talk about these thing and see how we can succeed, people in pharmacy people in the lab. So the team is not very large but we different parts doing different parts at the same time those different parts are moving as well, any other contribution, I would want to hear from you if there is any other contribution with what I have mentioned. How about at the point of care, in terms of having testing everyone who comes, so upon entry we are providing point of care testing so that anyone who comes get tested, I want to hear from you about how you think about this particular approach, is it a feasible approach so that everyone who comes for ART, they are also tested right there and then also OPD.**

R: I think it is good idea but the only thing is we end up, I don’t think, is it wasting resources? Yeah on some clients who don’t really need it at some point. Yeah. You find that whoever comes is test and at the end of the day we run out of test kits when we receive clients that need them. Maybe after they are doing screening maybe those who need to be tested then they are referred to point of care.

**I: Okay so my colleague here is saying that if we are testing everyone upon entry, we are somehow wasting resources. Because sometimes maybe you testing for BP it’s not there right, it means you could have wasted yeah. So how then do we select who we test? Should we have a strategy in place to say okay, this particular individual should test and this one should not, how do we, I know because long time ago it was test everyone who comes to the facility for convid-19 right? Then test kits run out, now we were like okay, people who are being tested are those with.**

R: with symptoms right

**I: With symptoms right? so it’s like you are wasting resources you test me am negative next time I come am negative, you have wasted two test kits right, I ow it to people that need to be tested, I want to find out in terms what your advise is, what strategy we can use to ensure that we are not wasting resources but that we test are those we might find with NCD so that we don’t waste the extra maybe test kits?**

R: Okay, basically from OPD what we use is that those with symptoms, for example HIV, symptoms pointing to that someone with a risk you also categorize them in that area, even if they are not having symptoms but there’s a risk to say this one probably could have gotten the disease we also put them their so that they are tested.

**I: Alright, thank you very much for that suggestion, any other suggestion?**

R: that’s why I think training is cardinal so that you even whoever is attending to that recipient of care is aware of who need to be tested because even HIV for example came with everybody being tested and now it ended up in wastage now we have a screening tool, it’s not everyone who should be tested and now in this screening tool there are counsellors and everyone is trained to screen who is supposed to be tested so training is cardinal.

**I: training is cardinal and also we need to have a screening tool, some tool that that will tell us who is now eligible to be tested right? Thank you very much, any other suggestion? so my last question will be, how do we know that integration is happening, how are we to know that it’s actually happening, how do we report that it is happening for example policy makers ask us okay now you are doing HIV integration right, how is happening, how are you knowing that it is happening because you might say by mouth to say you are providing care holistically and it’s so happening I come as a provider you are seated pointing there HIV here BP, how are we going to know of that integration is taking place if we say right now we are going to provide care in that manner how are we going to know?**

R: I think the report, either weekly or monthly; we say maybe how many clients were attended to for the past month and how many had form of high bloody pressure, I think the report.

**I: You are saying we need to have a report system right, whether weekly or monthly we need to report these numbers in terms of who, how many people had both conditions and things like that alright, I thing for me that I wanted to hear and to validate the results so I have validated myself by presenting what I have said, I did no lie anywhere right?**

R: group laughs

**I: (laughs) so most the time researchers have got the way of; they want to go, how can I say it? They want to make sure that the ideas they had are same ideas you have and in the report they will say no so they said no come back we lunch together and say this is what you said, do you have any other suggestion? in my closing remarks I will around the room to us each one of you to comment or say something whether the results are true or false or you are suggesting we do this and that in the future, whatever comment you want to say and I will ask the in charge to give us closing remarks and also say something. I know there are lot of things we have to so I thought let’s try and make this discussion short. So what I want to do first of all is to thank you, the first time we came we were told to go to whom we could talk to we completed our work. We went to analyse data, we came back again learn, and we thank you again for sharing this time teaching us every opportunity that you go through. So thank you for your time and we hope that we interact often, you will be seeing us again at some point when we come back because I think our work and your work toward ensuring that there’s care, the best care is given to the people that we see on daily bases yeah, I think those were my remarks. I will start from you I will go round until we reach there then the sister will close for us, so you can start.**

R: I would say the findings are correct so we just hope that, to see the implementation of the program and we have noted to say that the implementation will be effective if the supplies are made available, that what I wanted to say.

**I: Okay thank very much, next.**

R: The results were truly representation of what we have discussed.

**I: Okay thank very much.**

R: When are we starting is this just a meeting or we need to start?

**I: Okay, so what we did is to say let’s start; we try out with Chilienje and Mtendere for six months, after that we can enroll 15 facilities at the end of the year and do that for the next two years.**

R: So we can start tomorrow?

**I: we are going to be here as soon as possible (Laugh) my colleague here is ready to start as soon as possible**.

R: (group laughs) yeah because am ART and I know what is going on.

**I: What I going on right, so we will be here as soon to present the result go back change few things and come back again and then roll out**.

R: Thank you.

**I: Alright, thank you very much.**

R: The results are okay just as they have been projected.

**I: Alright, thank you very much, so hear my colleague from OPD.**

R: I think the results are okay we just hope you will be able to work with ministry of health so that as you said when we plan to stop this program, this program should continue.

**I: Alright, thank very much.**

R: I think the project is welcome we only hope it will start soon.

**I: Alright, thank you very much.**

R: We welcome the program and we just pray that it will start so that our friends they lessen that work especially for OPD since that the NCD will be shifted to ART.

**I: Okay thank you very Much.**

R: The project is welcome, we just want to see it continue after the project go so that the care continues.

**I: Okay thank you very much.**

R: we thank you for this project you are bringing to us we pray that we will work well together.

**I: Thank you very much.**

R: Thank you so much for this project, though is something which is already happening looking forward for simple report in templet, I don’t know whether it will be monthly or weekly yes.

R: I’m looking forward for the integration NCD from the ART.

**I: Okay thank you very much.**

R: thanks for the project we promise to work harder.

**I: Okay thank very much. So we already have the champion here who promises to work hard.**

R: The programme is welcome otherwise we are ready to support the project working hand in with our fellow staff workers.

**I: Okay, thank you very much.**

R: I would say the program seems to be projected to enhance service delivery which I think it will probably be achieved.

**I: Okay, thank you very much, my colleague behind there.**

R: I would like to say, we thank you so much for choosing Mtendere to be part of this project and we are hoping that we attain a good train of staffs life of these commodities.

**I: Thank you very much, yes my brother.**

R: Okay, I would say it’s a very good move, we are hoping that integration among can continue even if the program comes to an end.

**I: To continue isn’t, okay thank you very much, I will now hand over to the sister in charge its only good to have the boss because you pass on you don’t have to do everything yourself.**

R: Thank you very much; we really appreciate for this opportunity it’s rare event where you can sit and share ideas and definitely I appreciate you for coming it’s not easy abandoning what you were doing in your various departments but you came so that can rehearse one or two things so that we work together as a team this is capacity building and we really appreciate this project and am sure we are going to implement it without fail and it will be 100% actually. So what we need to work on is the reporting tool if it’s on a weekly bases we report is it monthly bases we need to hear from you, definitely we can report this on a monthly bases like the way we do with other reports, so for ART, this program is not only for ART, ART in charge is hear as you have seen different departments we receive different patients, the same patient that will go to ART, they may be find themselves in MCH, in Labour ward and also finally for the pharmacist. So I’m sure we are going to promise you as Mtendere health centre that we have received this project and we are not going to fail you we are going to implement it and also its one way of learning new things and we have learnt a lot this knowledge will definitely be shared with those people who are not here so that it can be worked upon and implement it in a good way. We really appreciate you for your coming thank you very much and we want to thank you for the food which is there at least after our talking we are going to gain the energy after wards. Thank you very much (group claps).

**END OF INTERVIEW**
